# Mood Disorders and Obstructive Sleep Apnea: A Systematic Review and Meta-Analysis

**DOI:** 10.3390/jcm15145478

**Published:** 2026-07-13

**Authors:** Buket Yeşiloğlu, Ümit Haluk Yeşilkaya, Deniz Bengi, Meltem Şen, Ceren Meriç Özgündüz, Bengisu Aksoy, Deniz Ceylan, Yüksel Peker

**Affiliations:** 1 Koç University Research Center for Translational Medicine, Koç University, 34010 Istanbul, Türkiye; byesiloglu23@ku.edu.tr (B.Y.); ceren.ozgunduz@acibadem.edu.tr (C.M.Ö.); baksoy24@ku.edu.tr (B.A.); dozalp@ku.edu.tr (D.C.); 2 Department of Neuroscience, Graduate School of Health, Koç University, 34010 Istanbul, Türkiye; 3 Department of Neurology, Division of Sleep Medicine, Beth Israel Deaconess Medical Center, Boston, MA 02215, USA; uyesilka@bidmc.harvard.edu; 4 Division of Sleep Medicine, Harvard Medical School, Boston, MA 02215, USA; 5 Neuroscience Graduate Program, Department of Psychiatry and Behavioural Neurosciences, McMaster University, Hamilton, ON L8P 0A6, Canada; bengid@mcmaster.ca; 6 Mood Disorders Treatment and Research Centre, Women’s Health Concerns Clinic, St. Joseph’s Healthcare Hamilton, Hamilton, ON L9C 0E3, Canada; 7 Department of Psychiatry, Kahta Governmental Hospital, 02400 Adıyaman, Türkiye; drmeltemsen@gmail.com; 8 Department of Psychiatry, School of Medicine, Acıbadem University, 34750 Istanbul, Türkiye; 9 Department of Psychiatry, School of Medicine, Koç University, 34010 Istanbul, Türkiye; 10 Department of Psychiatry & Psychology, Mayo Clinic, Rochester, MN 55905, USA; 11 Department of Pulmonology, Faculty of Medicine, Koç University, 34010 Istanbul, Türkiye; 12 Department of Molecular and Clinical Medicine, Institute of Medicine, Sahlgrenska Academy, University of Gothenburg, 40530 Gothenburg, Sweden; 13 Department of Clinical Sciences, Respiratory Medicine and Allergology, Faculty of Medicine, Lund University, 22100 Lund, Sweden; 14 Division of Pulmonary Allergy and Critical Care Medicine, University of Pittsburgh Medical Center, Pittsburgh, PA 15213, USA

**Keywords:** obstructive sleep apnea, depression, bipolar, mood

## Abstract

**Background/Objectives:** Mood disorders, such as major depressive disorder (MDD) and bipolar disorder (BD), along with obstructive sleep apnea (OSA), are prevalent conditions that have serious effects on affected individuals and public health. These issues have received more attention recently, as research shows there are similar risk factors and they can influence each other by worsening symptoms and treatment response. However, there is still no clear understanding of the extent of this co-occurrence and the factors that influence it. **Methods**: A systematic review of comorbid OSA and mood disorders was conducted from EMBASE, Ovid MEDLINE, Global Health, and APA PsycINFO (1 January 2013–25 July 2025), with meta-analyses applying random-effects models in R (metafor). Heterogeneity, publication bias, subgroup effects, and meta-regression analyses were performed by assessing demographic, clinical, and study quality variables. **Results**: Out of 6221 screened studies, 23 articles met eligibility criteria, yielding 2,380,986 OSA patients, with 446,495 comorbid mood disorders (MDD = 446,290; BD = 205), and 5724 mood disorder patients (MDD = 1987; BD = 3737) with 450 comorbid OSA. All meta-analyses demonstrated a significant prevalence of comorbidity, accompanied by substantial heterogeneity. Meta-regression analyses identified mean age (β = 0.18, 95% CI 0.03–0.33, *p* = 0.02) and mean body mass index (β = −0.18, 95% CI −0.32 to −0.04, *p* = 0.01) as significant moderators of OSA prevalence. **Conclusions**: This meta-analysis indicates a high prevalence of comorbidity between OSA and mood disorders, including MDD and BD, with significant heterogeneity. Age and body mass index emerged as significant moderators of OSA prevalence, emphasizing the need for systematic screening and tailored approaches in both clinical practice and research.

## 1. Introduction

Mood disorders, such as major depressive disorder (MDD) and bipolar disorder (BD), are highly prevalent and disabling disorders that are characterized by chronic mood and energy swings. Sleep plays a significant role as a core clinical feature and a trigger of illness onset while opposing clinical course [1,2,3,4,5]. Obstructive sleep apnea (OSA) is a highly common sleep disorder that affects nearly one billion adults worldwide [6]. It is characterized by recurrent upper airway obstruction during sleep, which is a relevant yet underrecognized comorbidity in mood disorders [7,8].

Considering this framework, recent systematic reviews and meta-analyses have shifted attention to the coexistence of OSA and mood disorders. Existing literature demonstrates that individuals with OSA have a higher risk of developing MDD, while patients with post-traumatic stress disorder and MDD show increased rates of OSA compared with healthy controls [9,10]. In addition, OSA in BD patients exhibited an elevated incidence of comorbidity, with male sex as a significant predictor [11]. Other reviews emphasize that untreated OSA may worsen mood symptoms and cognitive functioning, which underlines the clinical importance of recognizing and treating OSA rather than considering sleep-related symptoms as a psychiatric illness or its treatment [12,13].

Besides comorbidity, OSA and mood disorders share common clinical features with impaired energy, concentration, and cognitive functioning. Moreover, they frequently co-occur in shared metabolic vulnerability, including obesity, diabetes, and metabolic syndrome [2,3,14,15]. Additionally, both conditions are independently associated with negative long-term outcomes, such as cardiovascular disease, cognitive decline, dementia risk, and premature mortality [6,7,8,16]. These similar clinical and prognostic features suggest a bidirectional and potentially interacting relationship that raises the possibility of shared biological mechanisms. Besides growing epidemiological evidence, the size of this comorbidity and the factors causing heterogeneity across studies remain incompletely understood.

Accordingly, the present study aims to conduct a comprehensive systematic review and meta-analysis to quantify the prevalence of OSA in mood disorders and vice versa, and to explore potential sources of heterogeneity through subgroup and meta-regression analyses.

## 2. Materials and Methods

### 2.1. Literature Search

This systematic review has been registered in PROSPERO (Registration ID: CRD42023445215) and has been conducted according to the Preferred Reporting Items for Systematic Reviews and Meta-Analyses (PRISMA) statement [17], provided as Supplementary Table S1. A literature search was conducted in two parts: from January 2013 to May 2023 and from May 2023 to 25 July 2025. The electronic databases of EMBASE, Ovid MEDLINE, Global Health, and APA PsycINFO were used for the literature search with the keywords [(mood) OR (mood disorder) OR (mani*) OR (depres*) OR (bipolar)] AND [(sleep-related breathing disorder*) OR (obstructive sleep apnea) OR (OSA) OR (sleep apnea)].

### 2.2. Study Selection

The identified articles from the first part were uploaded to Rayyan (Rayyan Systems Inc., Cambridge, MA, USA; available at https://www.rayyan.ai/; accessed on 16 May 2023), while the second part was conducted wholly through Covidence (Veritas Health Innovation Ltd., Melbourne, Australia; available at https://www.covidence.org/; accessed on 10 July 2026). Rayyan was only used for title and abstract screening in the first part. Later, selected articles were imported to Covidence along with the new articles from the second part for the remaining steps of the systematic review. All steps were performed by two independent reviewers, with a third reviewer resolving conflicts and cross-checking all steps to prevent missing information. Citations of relevant articles, as well as the reference list of the included articles and reviews, were examined to identify overlooked studies. Original research articles were included in this review if they met the following criteria: (i) observational studies, (ii) including patients diagnosed with MDD or BD according to the DSM criteria or ICD classification systems, as well as clinician-administered validated assessment methods, (iii) including the OSA diagnosis through objective testing methods, (iv) studies that were conducted with a cohort of adults (age ≥ 18), (v) studies with a comparative group, (vi) studies that were written in English, and (vii) published between 1 January 2013 and 25 July 2025.

The quality assessment tool in Covidence was customized to the Modified Newcastle–Ottawa Quality Assessment Scale for case–control studies to evaluate the risk of bias in the included studies. The assessment of this scale is based on three criteria: (1) selection, (2) comparability, and (3) exposure factors. Each article was evaluated by two reviewers independently, with a third resolving conflicts, yielding scores of 0 (lowest) to 8 (highest) in total. The studies were ranked as low, moderate, and high quality with scores 0–2, 3–5, and 6–8, respectively.

### 2.3. Statistical Analysis

All statistical analyses were performed using R 4.5.1 (R Foundation for Statistical Computing, Vienna, Austria) with the metafor package (version 4.8-0). Meta-analyses were conducted to estimate the pooled prevalence of comorbid mood disorders among OSA patients (mood disorders in OSA) as well as comorbid OSA among individuals with mood disorders (OSA in mood disorders). All analyses were held for two analytical directions: (1) mood disorders in OSA, (2) OSA in mood disorders.

Prevalence for each population was calculated as the number of comorbid individuals divided by the total number of the reference group. The inverse-variance method with a random-effects model using restricted maximum likelihood (REML) was applied in meta-analyses due to expected heterogeneity across studies. Proportions were converted by logit transformation (PLOGIT) to stabilize variances and process extreme values of prevalence appropriately. Results were transformed back to pooled prevalence estimates with 95% confidence intervals (CIs). Cochran Q statistic, quantified with I^2^, was used to evaluate heterogeneity. I^2^ results were determined to represent low (25%), moderate (50%), and high heterogeneity (75%). Forest plots were generated using logit-transformed prevalence estimates to visualize the findings of each analysis. Publication bias was evaluated by funnel plots visually and Egger’s regression test statistically, when there were at least 10 studies.

To identify potential sources of heterogeneity, subgroup analyses were performed with the groups of (1) mood disorders subtype, (2) diagnostic tool for mood disorders, and (3) diagnostic method for OSA. For each subgroup analysis, separate random-effects meta-analyses were used. These analyses were held independently for both analytical directions and were reported as pooled prevalence estimates with 95% CIs.

To further investigate the heterogeneity of study-level characteristics, meta-regression analyses were conducted on the moderators of (1) female participant percentage, (2) mean age, (3) mean BMI, (4) mean Apnea–Hypopnea Index (AHI) scores, and (5) NOS scores, individually. Results of meta-regression were reported as regression coefficients (β) with corresponding 95% CIs and *p*-values.

In all statistical tests, a *p*-value < 0.05 was considered statistically significant.

## 3. Results

### 3.1. Study Selection Results

The first part consists of the articles published between January 2013 and May 2023. These articles were uploaded to Rayyan for title and abstract screening. In total, 4836 studies were imported; 1935 duplicates were eliminated, and 2901 studies were screened. Out of these studies, 31 were eligible for the next step. These studies were uploaded to Covidence for further steps.

**Figure 1:**
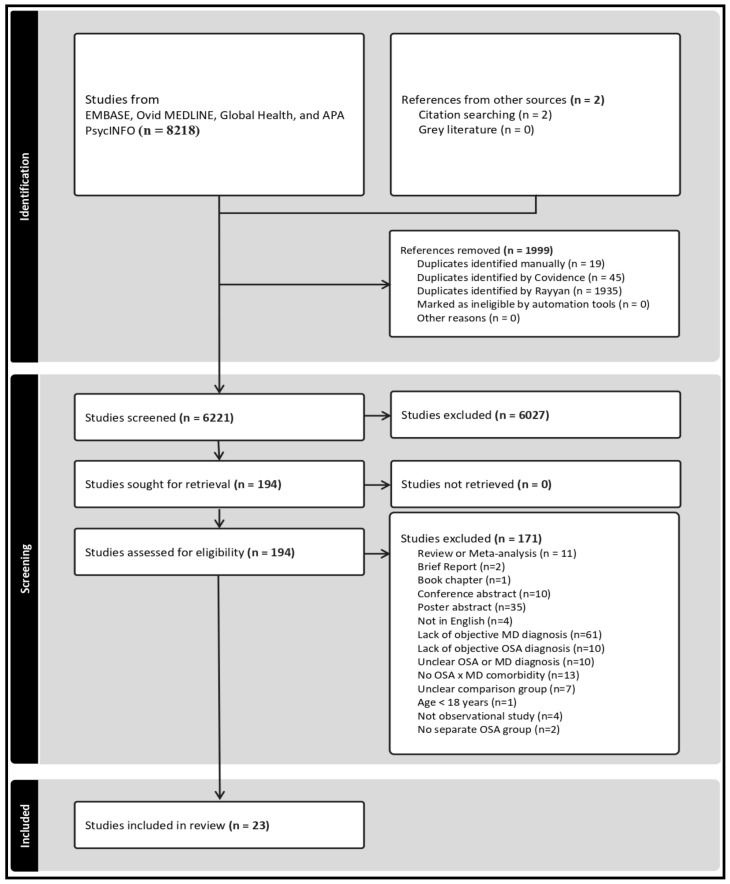
PRISMA flow diagram.

In the second part of the study, studies published from May 2023 to July 2025 were uploaded to Covidence, along with 31 additional eligible articles from the first part. A total of 3382 studies were imported for screening, along with 64 duplicates. After the duplicate removal, 3318 were screened for the first step, resulting in 194 studies for full-text screening. Out of 194, 23 were included in the systematic review. Two of these [18,19] reported the same population results. Hence, these two studies were merged into one study. Finally, 22 independent study populations were reviewed. The excluded studies and reasons for exclusion are listed in Supplementary Table S2. Figure 1 summarizes the whole literature search as a PRISMA flow diagram.

### 3.2. Study Characteristics

Among the 22 included articles, 12 were retrospective cohort [20,21,22,23,24,25,26,27,28,29,30,31], seven were cross-sectional [19,32,33,34,35,36,37], two were prospective cohort [38,39], and one study was a randomized clinical trial [40]. Thirteen investigated mood disorders comorbidity in OSA patients [19,22,23,24,25,28,29,31,32,33,34,35,36], while nine studied the mood disorders population with OSA [20,26,27,30,37,38,39,40]. In addition, 15 included patients with MDD [19,21,22,24,26,27,30,31,32,33,34,35,36,39,40], two had individuals with BD [37,38], while five had both MDD and BD patients [20,23,25,28,29]. The diagnostic tool of mood disorders was also varied in studies, as 10 used DSM/SCID [20,22,25,26,27,33,35,37,39,40], nine applied ICD [21,24,28,29,30,31,32,36,38], while three administered MINI [19,23,34] for mood disorders diagnosis. Moreover, there are 19 studies that used PSG [19,20,21,22,23,25,26,27,28,30,32,33,34,35,36,37,38,39,40], while three administered ICD [24,29,31] to diagnose OSA. Quality assessment results of the studies were mostly high, with two moderate-scoring studies [23,34]. The results of the literature search are summarized in Table 1.

### 3.3. Mood Disorder Prevalence in the OSA Population

To ensure appropriate meta-analytic comparisons, studies including both MDD and BD were split by mood disorder type. As a result, there were 17 different groups derived from 13 studies in all the analyses.

**Figure 2:**
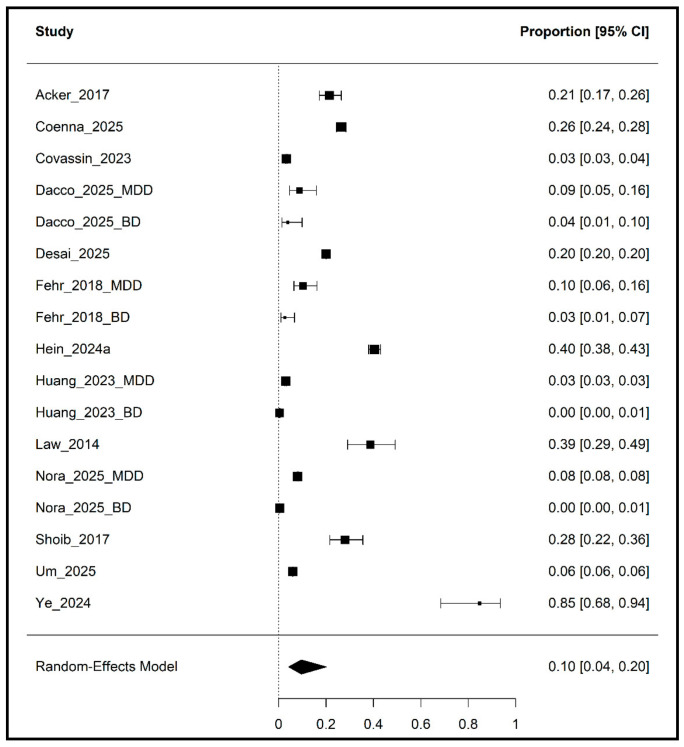
Forest plot of the mood disorder prevalence in the OSA population [19,22,23,24,25,26,28,29,31,32,34,35,36].

A random-effects meta-analysis was performed to calculate the estimated pooled prevalence of mood disorders among individuals with OSA. In total, 2,380,986 OSA patients with 446,495 mood disorder comorbidities (MDD = 446,290 and BD = 205) were included in the meta-analysis for the mood disorder prevalence of OSA patients. As a result of the meta-analysis, the pooled prevalence of mood disorder was 10% (95% CI: 0.04–0.20). The forest plot of the analysis is shown in Figure 2. There was a significant heterogeneity across studies in the overall analysis (I^2^ = 99.90%, Q = 20,131.25, *p* < 0.001). There was no significant publication bias according to Egger’s regression-based test (*p* = 0.06). The funnel plot of the analysis is shown as Supplementary Figure S1.

### 3.4. Subgroup Analyses of Mood Disorder Prevalence in the OSA Population

Subgroup analysis based on mood disorder subtype was conducted for comorbid-MDD (13 studies) and comorbid-BD (four studies) patients. As a result, the pooled prevalence of MDD was 17% (95% CI: 0.09–0.30), while the pooled prevalence of BD was 1% (95% CI: 0.00–0.03). The forest plots of the analyses are shown in Supplementary Figures S2 and S3. There was a significant heterogeneity across studies with the MDD (I^2^ = 99.97%, Q = 16,980.87, *p* < 0.0001) and BD (I^2^ = 96.42%, Q = 30.68, *p* < 0.0001). Egger’s regression test for MDD indicated evidence of small-study effects (*p* = 0.035), which suggests possible publication bias. This test was not applicable for BD due to the small study size. The funnel plots of the analysis for both MDD and BD are shown as Supplementary Figures S4 and S5, respectively.

Subgroup analysis according to the mood disorder diagnostic tool was conducted for DSM/SCID (five studies), ICD (eight studies), and MINI (four studies). As a result, the pooled prevalence of DSM/SCID was 25% (95% CI: 0.06–0.65), the pooled prevalence of ICD was 4% (95% CI: 0.01–0.11), while the pooled prevalence of MINI was 16% (95% CI: 0.05–0.38). The forest plots of the analyses are shown in Supplementary Figures S6–S8. There was a significant heterogeneity across studies with the DSM/SCID (I^2^ = 99.72%, Q = 161.95, *p* < 0.0001), ICD (I^2^ = 99.99%, Q = 19,580.43, *p* < 0.0001), and MINI (I^2^ = 95.11%, Q = 39.11, *p* < 0.0001). Egger’s regression test was not applicable for any of the groups due to the small study size. The funnel plots of the analysis for DSM/SCID, ICD, and MINI are shown as Supplementary Figures S9, S10, and S11, respectively.

Subgroup analysis based on the OSA diagnostic method was conducted for PSG (13 studies) and ICD (four studies). As a result, the pooled prevalence of PSG was 12% (95% CI: 0.05–0.27), while the pooled prevalence of ICD was 5% (95% CI: 0.01–0.21). The forest plots of the analyses are shown in Supplementary Figures S12 and S13. There was a significant heterogeneity across studies with the PSG (I^2^ = 99.72%, Q = 3210.99, *p* < 0.0001) and ICD (I^2^ = 99.99%, Q = 16,309.56, *p* < 0.0001). There was no significant publication bias according to Egger’s regression-based test for PSG (*p* = 0.88). This test was not applicable for ICD due to the small study size. The funnel plots of the analysis for both PSG and ICD are shown as Supplementary Figures S14 and S15, respectively.

### 3.5. OSA Prevalence in the Mood Disorder Population

For appropriate meta-analytic comparisons, studies including both MDD and BD were also split by mood disorder type in this analytic direction. As a result, there were 10 different groups derived from nine studies in all the analyses.

**Figure 3:**
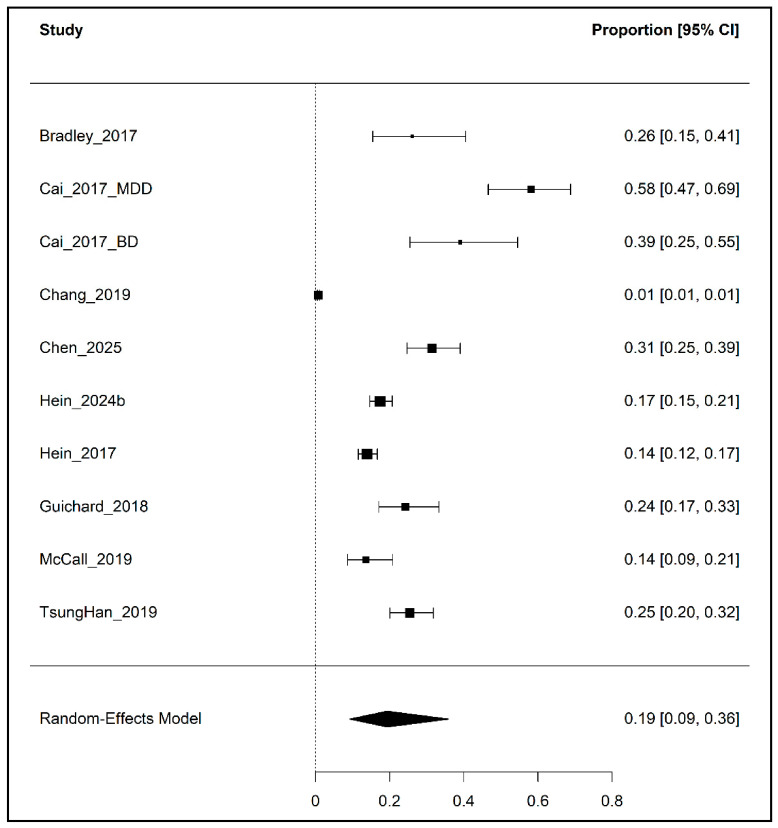
Forest plot of the OSA prevalence in the mood disorder population [20,21,27,30,33,37,38,39,40].

A random-effects meta-analysis was conducted for the estimated pooled prevalence of OSA among individuals with mood disorders. In total, 5724 (MDD = 1987 and BD = 3737) mood disorder patients with 450 comorbid OSA were included in the meta-analysis for the OSA prevalence of mood disorder patients. As a result of the meta-analysis, the pooled prevalence of OSA was 19% (95% CI: 0.09–0.36). The forest plot of the analysis is shown in Figure 3. There was a significant heterogeneity across studies in the overall analysis (I^2^ = 98.34%, Q = 422.08, *p* < 0.0001). There was no significant publication bias according to Egger’s regression-based test (*p* = 0.44). The funnel plot of the analysis is shown as Supplementary Figure S16.

### 3.6. Subgroup Analyses of OSA Prevalence in the Mood Disorder Population

Subgroup analysis based on mood disorder subtype was held for OSA with MDD (seven studies) and BD (three studies) patients. As a result, the pooled prevalence of MDD was 24% (95% CI: 0.16–0.36), while the pooled prevalence of BD was 11% (95% CI: 0.01–0.64). The forest plots of the analyses are shown in Supplementary Figures S17 and S18. There was a significant heterogeneity across studies with the MDD (I^2^ = 95.28%, Q = 91.73, *p* < 0.0001) and BD (I^2^ = 98.67%, Q = 192.11, *p* < 0.0001). Egger’s regression test was not applicable for MDD nor BD due to the small study size. The funnel plots of the analysis for both MDD and BD are shown as Supplementary Figures S19 and S20, respectively.

Subgroup analysis according to the mood disorder diagnostic tool was conducted for DSM/SCID (seven studies) and ICD (three studies). As a result, the pooled prevalence of DSM/SCID was 25% (95% CI: 0.16–0.38), while the pooled prevalence of ICD was 10% (95% CI: 0.01–0.58). The forest plots of the analyses are shown in Supplementary Figures S21 and S22. There was a significant heterogeneity across studies with the DSM/SCID (I^2^ = 94.47%, Q = 84.85, *p* < 0.0001) and ICD (I^2^ = 99.43%, Q = 313.29, *p* < 0.0001). Egger’s regression test was not applicable for any of the groups due to the small study size. The funnel plots of the analysis for DSM/SCID and ICD are shown as Supplementary Figures S23 and S24, respectively.

Subgroup analysis based on the OSA diagnostic method was not conducted since all the studies in this analytic direction performed PSG to diagnose patients.

All results of the analyses are given as Supplementary Table S3.

### 3.7. Meta-Regression Analyses

Meta-regression analyses were performed to investigate potential moderators for heterogeneity. There were no significant associations for sex, mean age, mean BMI, mean AHI scores, or NOS scores in mood disorder prevalence (all *p* > 0.05). On the other hand, the mean age (β = 0.18, 95% CI 0.03–0.33, *p* = 0.02) and the mean BMI (β = −0.18, 95% CI −0.32 to −0.04, *p* = 0.01) were observed to have significant associations in OSA prevalence. According to the findings, OSA prevalence increases with higher mean age, while mean BMI has the opposite association with OSA prevalence.

All results of the meta-regression analyses are given as Supplementary Table S4.

## 4. Discussion

This systematic review and meta-analysis revealed that OSA and mood disorders co-occur in both directions, mood disorders in OSA and OSA in mood disorders, even though the magnitude of this association differs depending on the diagnostic perspective. Mood disorders were present in approximately 10% of individuals with OSA, whereas OSA was identified in about 19% of individuals with mood disorders, pointing to an asymmetrical relationship. In contrast to earlier meta-analyses, we examined both directions of association and considered MDD and BD separately. Across analysis, MDD was more common than BD. High heterogeneity was observed among all analyses, while age and BMI were detected as potential variation sources in OSA prevalence, suggesting the contribution of demographic and metabolic factors.

Findings of the present study are consistent with previous reviews reporting increased prevalence of depressive symptoms [41] and diagnosed mood disorders with OSA [42,43]. Asymmetry in prevalence estimates was observed, with a lower pooled prevalence of mood disorders in OSA patients compared with a higher prevalence of OSA in mood disorder patients. This may suggest differential diagnostic recognition across clinical settings. Mood disorders are usually unrecognized outside psychiatric care, especially in sleep clinic populations. Contrarily, individuals with mood disorders that have prominent sleep-related and somatic complaints usually undergo OSA evaluation. Independent of the underlying biological mechanism, this asymmetry might occur due to differences in symptom visibility, help-seeking methods, the episodic nature of mood disorders, and stigma-related barriers.

Compared with BD, MDD demonstrated higher pooled prevalence estimates in both analytical directions, consistent with previous findings [43]. The reasons behind the higher prevalence of OSA in individuals with MDD might be sleep fragmentation related to hypoxemia, alterations in serotonergic neurotransmission, and shared somatic risk factors, including obesity, cardiovascular disease, and metabolic dysregulation [44,45,46,47]. In contrast, the lower prevalence of BD in OSA patients may reflect a combination of underdiagnosis in sleep clinic settings, phenotypic overlap in which depressive symptoms are subsumed within the clinical presentation of OSA, age-related differences in disorder onset, and referral biases between the two mood disorders. The episodic and history-dependent nature of BD, together with stigma-related diagnostic barriers, may further contribute to its lower observed prevalence relative to MDD. Nevertheless, these findings should be interpreted with caution, as the BD analyses were based on a relatively small number of studies and participants compared with those for MDD. Consequently, the pooled estimates for BD are less precise and should be considered preliminary until confirmed by larger, well-designed studies.

Notably, subgroup analyses according to diagnostic methodology demonstrated that studies using structured psychiatric diagnostic interviews (DSM/SCID or MINI) generally reported higher pooled prevalence estimates than those relying on administrative ICD-based diagnoses in both analytical directions. Similarly, studies diagnosing OSA using PSG reported higher pooled prevalence estimates than those using administrative ICD-based diagnoses. These findings suggest that differences in diagnostic ascertainment may have contributed to the observed variability across studies. Structured clinical interviews and PSG provide more comprehensive and standardized diagnostic assessment, whereas administrative ICD coding relies on healthcare utilization and coding practices and may underestimate the true prevalence of mood disorders and OSA. Therefore, the pooled prevalence estimates should be interpreted in the context of the diagnostic methods used by the included studies. The higher prevalence estimates observed in studies using structured psychiatric interviews and PSG suggest that reliance on administrative diagnostic codes may underestimate the true burden of comorbidity, while differences in diagnostic methodology may partly explain the substantial heterogeneity observed across analyses.

In all analyses, heterogeneity was too high (I^2^ > 95%), underlying the complex association between mood disorders and OSA. Even after subgroup analyses in the mood disorder subtype (MDD or BD), heterogeneity persisted. To identify potential sources of heterogeneity, meta-regression was performed. However, there were no moderators—including sex distribution, age, BMI, AHI, or study quality—that significantly explained the estimated prevalence variance in the mood disorders with comorbid OSA. On the other hand, mean age and BMI demonstrated a significant association with OSA prevalence. These findings are consistent with a previous meta-analysis that investigated OSA prevalence in MDD, BD, and schizophrenia [43]. According to these findings, demographic factors might contribute to OSA prevalence in individuals with mood disorders. However, the inverse association observed between BMI and OSA prevalence should be interpreted cautiously, as it appears inconsistent with the well-established role of obesity as one of the strongest risk factors for OSA in the general population. First, the meta-regression was based on a limited number of studies reporting BMI, reducing the stability and generalizability of the estimated association. Second, one of the included studies [20] applied a BMI-based inclusion criterion (BMI ≤ 30 kg/m^2^), which may have restricted the BMI distribution of the study population and influenced the observed study-level association. Finally, because meta-regression was performed using study-level mean BMI rather than individual participant data, the analysis is susceptible to ecological bias and cannot be interpreted at the individual level. Therefore, the observed inverse association is more likely to reflect differences in study populations, participant selection, and methodological characteristics across studies than a true biological relationship. However, they are not the main reason for the heterogeneity across studies. The high remaining heterogeneity suggests that the pooled prevalence estimates should be viewed as general summaries of the overall burden of comorbidity, rather than exact figures that apply to every clinical setting. This variation is likely due to differences in study populations, recruitment settings, diagnostic methods, and clinical characteristics that could not be fully captured in our analyses. However, despite these differences in prevalence estimates, the consistent finding of a bidirectional relationship across studies highlights the clinical importance of routinely screening individuals with OSA for mood disorders, and vice versa. In other words, while the exact prevalence may differ between populations, the association itself appears to be consistent and clinically meaningful.

Despite conducting a comprehensive systematic review and rigorous statistical analyses with the use of subgroup and meta-regression analyses, there are several important limitations. First, the study consists of observational studies that can provide associations between mood disorders and OSA rather than causal relationships. Second, significant heterogeneity across analyses could not be fully explained, even though subgroup and meta-regression analyses were conducted to explore sources of variability, resulting in limited precision of the pooled estimates. Another important limitation is the limited availability of detailed clinical information across the included studies. Most studies did not report OSA severity, treatment status (e.g., continuous positive airway pressure [CPAP] use or adherence), obesity severity, or OSA phenotypes in sufficient detail to allow stratified analyses. These factors may influence both the prevalence and clinical presentation of mood disorders and OSA and may therefore have contributed to the residual heterogeneity observed in our analyses. Future studies should report these characteristics more consistently to enable more detailed investigation of potential sources of variability. Finally, the relatively small number of studies and participants available for certain subgroups, particularly BD, limits the precision and generalizability of these subgroup-specific findings. Therefore, conclusions regarding BD should be interpreted with caution until additional studies provide more robust evidence.

## 5. Conclusions

The findings of this systematic review and meta-analysis indicate a strong bidirectional association between OSA and mood disorders, especially MDD, highlighting the importance of routine sleep-related breathing disorder screening in psychiatric populations, as well as screening mood disorder comorbidities in sleep-related disorder populations. Finally, the persistent heterogeneity across studies underlines the need for well-designed longitudinal studies using standardized and detailed diagnostic criteria to clarify causal mechanisms and inform more effective intervention strategies.

## Figures and Tables

**Table 1:** Characteristics of included studies.

No. of Study	Study ID	Study Type	Type of Mood Disorder	No. of Total	No. of Comorbid	No. of Standalone	Female (%)	Age (Mean)	BMI (Mean)	Mood Disorder Diagnosis	OSA Diagnosis	AHI (Mean)	Quality Assessment
**Mood disorder prevalence in the OSA population**													
1	Acker et al., 2017 [36]	CS	MDD	303	65	238	NR	57.5	NR	ICD	PSG	31.2	6
2	Conenna et al., 2025 [22]	RC	MDD	1849	487	1362	26.1	51.00	29.00	DSM/SCID	PSG	15	7
3	Covassin et al., 2023 [32]	CS	MDD	14,823	495	14,328	38.7	61.00	32.60	ICD	PSG	17	6
4	Dacco et al., 2025 [23]	RC	MDD	103	9	94	37.41	38	30.105	MINI	PSG	NR	5
5	Dacco et al., 2025 [23]	RC	BD	103	4	99	37.41	38	30.105	MINI	PSG	NR	5
6	Desai et al., 2025 [24]	RC	MDD	2,169,730	435,185	1,734,545	55.35	64.5	NR	ICD	ICD	NR	7
7	Fehr et al., 2018 [25]	RC	MDD	155	16	139	9.03	59	32.43	DSM/SCID	PSG	NR	6
8	Fehr et al., 2018 [25]	RC	BD	155	4	151	9.03	59	32.43	DSM/SCID	PSG	NR	6
9	Hein et al., 2024 [26]	CS	MDD	1488	602	886	23.0	51	29.00	DSM/SCID	PSG	14	7
10	Huang et al., 2023 [28]	RC	MDD	7722	228	7494	44.74	57.89	NR	ICD	PSG	NR	7
11	Huang et al., 2023 [28]	RC	BD	7722	29	7693	44.74	57.89	NR	ICD	PSG	NR	7
12	Law et al., 2014 [34]	CS	MDD	88	34	54	29.7	49.2	33.4	MINI	PSG	14.5	5
13	Nora et al., 2025 [29]	RC	MDD	36,385	2917	33,468	31.83	66	NR	ICD	ICD	NR	7
14	Nora et al., 2025 [29]	RC	BD	36,385	168	36,217	31.83	66	NR	ICD	ICD	NR	7
15	Shoib et al., 2017 [19]	CS	MDD	157	44	113	NR	54.89	31.09	MINI	PSG	21.78	6
16	Um et al., 2025 [31]	RC	MDD	10,3785	6180	97,605	16.94	NR	NR	ICD	ICD	NR	7
17	Ye et al., 2024 [35]	CS	MDD	33	28	5	12.12	42.15	27.69	DSM/SCID	PSG	43.46	6
**OSA prevalence in the mood disorder population**													
18	Bradley et al., 2017 [37]	CS	BD	46	12	34	67.4	46.8	30	DSM/SCID	PSG	5.3	7
19	Cai et al., 2017 [20]	RC	MDD	74	43	31	64.3	51.58	22.48	DSM/SCID	PSG	NR	7
20	Cai et al., 2017 [20]	RC	BD	41	16	25	64.3	51.58	22.48	DSM/SCID	PSG	NR	7
21	Chang et al., 2019 [38]	PC	BD	3650	28	3622	56.1	39.84	NR	ICD	PSG	NR	7
22	Chen et al., 2025 [21]	RC	MDD	159	50	109	59.7	46.00	22.59	ICD	PSG	3.0	7
23	Hein et al., 2024 [33]	RC	MDD	607	106	501	52.7	50	27.8	DSM/SCID	PSG	3.0	7
24	Hein et al., 2017 [27]	RC	MDD	703	98	605	54.48	44.96	27.3	DSM/SCID	PSG	2	7
25	Guichard et al., 2018 [39]	PC	MDD	107	26	81	79.4	56.73	27.22	DSM/SCID	PSG	14.81	6
26	McCall et al., 2019 [40]	RCT	MDD	125	17	108	63.2	41.7	28.15	DSM/SCID	PSG	5.34	7
27	Tsung-Han et al., 2019 [30]	RC	MDD	212	54	158	56.6	49.5	24.2	ICD	PSG	7.1	7

NR, not reported.

## Data Availability

Data collected for the study, including de-identified individual participant data, will be made available to others within 6 months after the publication of this article for academic purposes (e.g., meta-analyses), upon request to the first author (byesiloglu23@ku.edu.tr) and with a signed data access agreement.

## Supplementary Materials

The following supporting information can be downloaded at: https://www.mdpi.com/article/10.3390/jcm15145478/s1, Figure S1: Funnel plot of the mood disorder prevalence in the OSA population; Figure S2: Forest plot of MDD in mood disorder prevalence in the OSA population; Figure S3: Forest plot of BD in mood disorder prevalence in the OSA population; Figure S4: Funnel plot of MDD in mood disorder prevalence in the OSA population; Figure S5: Funnel plot of BD in mood disorder prevalence in the OSA population; Figure S6: Forest plot of DSM/SCID in mood disorder prevalence in the OSA population; Figure S7: Forest plot of ICD in mood disorder prevalence in the OSA population; Figure S8: Forest plot of MINI in mood disorder prevalence in the OSA population; Figure S9: Funnel plot of DSM/SCID in mood disorder prevalence in the OSA population; Figure S10: Funnel plot of ICD in mood disorder prevalence in the OSA population; Figure S11: Funnel plot of MINI in mood disorder prevalence in the OSA population; Figure S12: Forest plot of PSG in mood disorder prevalence in the OSA population; Figure S13: Forest plot of ICD in mood disorder prevalence in the OSA population; Figure S14: Funnel plot of PSG in mood disorder prevalence in the OSA population; Figure S15: Funnel plot of ICD in mood disorder prevalence in the OSA population; Figure S16: Funnel plot of the OSA prevalence in the mood disorder population; Figure S17: Forest plot of MDD in OSA prevalence in the mood disorder population; Figure S18: Forest plot of BD in OSA prevalence in the mood disorder population; Figure S19: Funnel plot of MDD in OSA prevalence in the mood disorder population; Figure S20: Funnel plot of BD in OSA prevalence in the mood disorder population; Figure S21: Forest plot of DSM/SCID in OSA prevalence in the mood disorder population; Figure S22: Forest plot of ICD in OSA prevalence in the mood disorder population; Figure S23: Funnel plot of DSM/SCID in OSA prevalence in the mood disorder population; Figure S24: Funnel plot of ICD in OSA prevalence in the mood disorder population; Table S1: PRISMA 2020 Checklists; Table S2: Excluded studies and exclusion reasons; Table S3: Results of meta-analyses; Table S4: Results of meta-regression analyses.
