# Supplementary material for: Mood Disorders and Obstructive Sleep Apnea: A Systematic Review and Meta-Analysis
Source: J Clin Med. 2026 Jul 13;15(14):5478. doi: 10.3390/jcm15145478 (PMC13413346; doi:10.3390/jcm15145478)
Supplement: Supplementary file 1 [file jcm-15-05478-s001.zip › jcm-4396568-supplementary.pdf]

Supplementary Materials

Supplementary Table S1: PRISMA Checklists

PRISMA 2020 for Abstracts Checklist

Section and Topic	Item #	Checklist item	Reported (Yes/No)
**TITLE**			
Title	1	Identify the report as a systematic review.	Y
**BACKGROUND**			
Objectives	2	Provide an explicit statement of the main objective(s) or question(s) the review addresses.	Y
**METHODS**			
Eligibility criteria	3	Specify the inclusion and exclusion criteria for the review.	N
Information sources	4	Specify the information sources (e.g. databases, registers) used to identify studies and the date when each was last searched.	Y
Risk of bias	5	Specify the methods used to assess risk of bias in the included studies.	N
Synthesis of results	6	Specify the methods used to present and synthesise results.	N
**RESULTS**			
Included studies	7	Give the total number of included studies and participants and summarise relevant characteristics of studies.	Y
Synthesis of results	8	Present results for main outcomes, preferably indicating the number of included studies and participants for each. If meta-analysis was done, report the summary estimate and confidence/credible interval. If comparing groups, indicate the direction of the effect (i.e. which group is favoured).	Y
**DISCUSSION**			
Limitations of evidence	9	Provide a brief summary of the limitations of the evidence included in the review (e.g. study risk of bias, in-consistency and imprecision).	Y
Interpretation	10	Provide a general interpretation of the results and important implications.	Y
**OTHER**			
Funding	11	Specify the primary source of funding for the review.	N
Registration	12	Provide the register name and registration number.	N

From: Page MJ, McKenzie JE, Bossuyt PM, Boutron I, Hoffmann TC, Mulrow CD, et al. The PRISMA 2020 statement: an updated guideline for reporting systematic re-views. BMJ 2021;372:n71. doi: 10.1136/bmj.n71. This work is licensed under CC BY 4.0. To view a copy of this license, visit https://creativecommons.org/licenses/by/4.0/

PRISMA 2020 Checklist

Section and Topic	Item #	Checklist item	Location where item is reported
**TITLE**			
Title	1	Identify the report as a systematic review.	Lines 2-3
**ABSTRACT**			
Abstract	2	See the PRISMA 2020 for Abstracts checklist.	Lines 33-55
**INTRODUCTION**			
Rationale	3	Describe the rationale for the review in the context of existing knowledge.	Lines 59-84
Objectives	4	Provide an explicit statement of the objective(s) or question(s) the review addresses.	Lines 85-88
**METHODS**			
Eligibility criteria	5	Specify the inclusion and exclusion criteria for the review and how studies were grouped for the syntheses.	Lines 107-113
Information sources	6	Specify all databases, registers, websites, organisations, reference lists and other sources searched or consulted to identify studies. Specify the date when each source was last searched or consulted.	Lines 94-96
Search strategy	7	Present the full search strategies for all databases, registers and websites, including any filters and limits used.	Lines 97-99
Selection process	8	Specify the methods used to decide whether a study met the inclusion criteria of the review, including how many reviewers screened each record and each report retrieved, whether they worked independently, and if applicable, details of automation tools used in the process.	Lines 101-108
Data collection process	9	Specify the methods used to collect data from reports, including how many reviewers collected data from each report, whether they worked independently, any processes for obtaining or confirming data from study investigators, and if applicable, details of automation tools used in the process.	Lines 103-106
Data items	10a	List and define all outcomes for which data were sought. Specify whether all results that were compatible with each outcome domain in each study were sought (e.g. for all measures, time points, analyses), and if not, the methods used to decide which results to collect.	N/A
	10b	List and define all other variables for which data were sought (e.g. participant and intervention characteristics, funding sources). Describe any assumptions made about any missing or unclear information.	N/A
Study risk of bias assessment	11	Specify the methods used to assess risk of bias in the included studies, including details of the tool(s) used, how many reviewers assessed each study and whether they worked independently, and if applicable, details of automation tools used in the process.	Lines 115-121
Effect measures	12	Specify for each outcome the effect measure(s) (e.g. risk ratio, mean difference) used in the synthesis or presentation of results.	Lines 128-129
Synthesis meth-ods	13a	Describe the processes used to decide which studies were eligible for each synthesis (e.g. tabulating the study intervention characteristics and comparing against the planned groups for each synthesis (item #5)).	N/A
	13b	Describe any methods required to prepare the data for presentation or synthesis, such as handling of missing summary statistics, or data conversions.	Lines 128-134
	13c	Describe any methods used to tabulate or visually display results of individual studies and syntheses.	Lines 136-138
	13d	Describe any methods used to synthesize results and provide a rationale for the choice(s). If meta-analysis was performed, describe the model(s), method(s) to identify the presence and extent of statistical heterogeneity, and software package(s) used.	Lines 128-134
	13e	Describe any methods used to explore possible causes of heterogeneity among study results (e.g. subgroup analysis, meta-regression).	Lines 134-137<br>Lines 140-149
	13f	Describe any sensitivity analyses conducted to assess robustness of the synthesized results.	N/A

Section and Topic	Item #	Checklist item	Location where item is reported
Reporting bias assessment	14	Describe any methods used to assess risk of bias due to missing results in a synthesis (arising from reporting biases).	Lines 137-139
Certainty assessment	15	Describe any methods used to assess certainty (or confidence) in the body of evidence for an outcome.	N/A
**RESULTS**			
Study selection	16a	Describe the results of the search and selection process, from the number of records identified in the search to the number of studies included in the review, ideally using a flow diagram.	Lines 153-166<br>Figure 1
	16b	Cite studies that might appear to meet the inclusion criteria, but which were excluded, and explain why they were excluded.	Lines 162-164
Study characteristics	17	Cite each included study and present its characteristics.	Lines 170-182<br>Table 1
Risk of bias in studies	18	Present assessments of risk of bias for each included study.	Table 1
Results of individual studies	19	For all outcomes, present, for each study: (a) summary statistics for each group (where appropriate) and (b) an effect estimate and its precision (e.g. confidence/credible interval), ideally using structured tables or plots.	Figure 2 & 3<br>Supplementary Figure S1-S24<br>Supplementary Table S3
Results of syntheses	20a	For each synthesis, briefly summarise the characteristics and risk of bias among contributing studies.	Table 1
	20b	Present results of all statistical syntheses conducted. If meta-analysis was done, present for each the summary estimate and its precision (e.g. confidence/credible interval) and measures of statistical heterogeneity. If comparing groups, describe the direction of the effect.	Lines 187-265
	20c	Present results of all investigations of possible causes of heterogeneity among study results.	202-230<br>247-275<br>Supplementary Table S3 & S4
	20d	Present results of all sensitivity analyses conducted to assess the robustness of the synthesized results.	N/A
Reporting biases	21	Present assessments of risk of bias due to missing results (arising from reporting biases) for each synthesis assessed.	Lines 197-263<br>Supplementary Figure S1-S24<br>Supplementary Table S3
Certainty of evidence	22	Present assessments of certainty (or confidence) in the body of evidence for each outcome assessed.	N/A
**DISCUSSION**			
Discussion	23a	Provide a general interpretation of the results in the context of other evidence.	Lines 287-322
	23b	Discuss any limitations of the evidence included in the review.	Lines 323-326
	23c	Discuss any limitations of the review processes used.	Lines 326-330
	23d	Discuss implications of the results for practice, policy, and future research.	Lines 331-337

Section and Topic	Item #	Checklist item	Location where item is reported
**OTHER INFORMATION**			
Registration and protocol	24a	Provide registration information for the review, including register name and registration number, or state that the review was not registered.	Lines 91-93
	24b	Indicate where the review protocol can be accessed, or state that a protocol was not prepared.	N/A
	24c	Describe and explain any amendments to information provided at registration or in the protocol.	N/A
Support	25	Describe sources of financial or non-financial support for the review, and the role of the funders or sponsors in the review.	Lines 346 & 351-353
Competing inter-ests	26	Declare any competing interests of review authors.	Line 357
Availability of data, code and other materials	27	Report which of the following are publicly available and where they can be found: template data collection forms; data extracted from included studies; data used for all analyses; analytic code; any other materials used in the review.	Lines 347-350

From: Page MJ, McKenzie JE, Bossuyt PM, Boutron I, Hoffmann TC, Mulrow CD, et al. The PRISMA 2020 statement: an updated guideline for reporting systematic reviews. BMJ 2021;372:n71. doi: 10.1136/bmj.n71. This work is licensed under CC BY 4.0. To view a copy of this license, visit https://creativecommons.org/licenses/by/4.0/

Supplementary Table S2: Excluded studies and exclusion reasons

Study ID	Reason
Abdella 2023	Not observational study
Alesci 2023	Lack of objective OSA diagnosis
Almagro 2024	Unclear OSA or MD diagnosis
Alzaabi 2025	Lack of objective MD diagnosis
Alzaabi 2023	Lack of objective MD diagnosis
Anderson 2024	No OSA x MD comorbidity
Appleton 2015	Lack of objective MD diagnosis
BaHammam 2023	Review
Bailer 2025	Unclear comparison group
Bailly 2024	Poster Abstract
Balasubramanian 2025	Lack of objective MD diagnosis
Baron 2024	Poster Abstract
Benca 2023	Review
Berk 2019	Lack of objective MD diagnosis
Best 2013	Unclear OSA or MD diagnosis
Bonfils 2023	No separate OSA group
Braham 2023	Conference abstract
Cambron-Mellott 2024	Poster Abstract
Campeanu 2023	Poster Abstract
Cangur 2024	Lack of objective MD diagnosis
Carneiro-Barrera 2023	Lack of objective MD diagnosis
Chai 2023	Poster Abstract
Cheng 2024	Poster Abstract
Cheng 2013	No OSA x MD comorbidity
Chen 2025	Lack of objective MD diagnosis
Chen 2023	Lack of objective MD diagnosis
Chen 2024	Unclear OSA or MD diagnosis
Chen 2023	Lack of objective MD diagnosis
Chen 2013	No OSA x MD comorbidity
Chen 2024	Conference abstract
Choi 2025	Lack of objective MD diagnosis
Chua 2023	Review
Chua 2025	Lack of objective MD diagnosis
CigdemKaracay 2023	Lack of objective MD diagnosis
Coelho 2025	Review
Cole 2024	Poster Abstract
Cole 2025	Poster Abstract
Cole 2025	Poster Abstract
Condoleo 2023	Lack of objective MD diagnosis
Correia 2023	Review
Cury 2025	Lack of objective MD diagnosis

Dacco 2025	Poster Abstract
DeAngelis 2023	Poster Abstract
deCenso 2024	Lack of objective MD diagnosis
deLima 2024	Lack of objective MD diagnosis
Delrosso 2024	Lack of objective MD diagnosis
deSouzaBezerra 2024	No OSA x MD comorbidity
DeWeerdt 2024	Lack of objective MD diagnosis
DeWeerdt 2025	Lack of objective MD diagnosis
Dharmage 2023	No OSA x MD comorbidity
Ditmer 2025	Lack of objective MD diagnosis
Doane 2024	Lack of objective OSA diagnosis
Doane 2023	Lack of objective OSA diagnosis
Douglas 2013	Lack of objective MD diagnosis
Drakatos 2023	Unclear OSA or MD diagnosis
Evans 2024	Lack of objective MD diagnosis
Evlampieva 2024	Not in English
Feng 2025	Review
Fki 2024	Poster Abstract
Frame 2024	Poster Abstract
Fridriksson 2023	Lack of objective MD diagnosis
Fuller-Rowell 2024	Lack of objective MD diagnosis
Furihata 2024	Lack of objective MD diagnosis
Furlan 2024	Poster Abstract
Furlan 2023	Poster Abstract
Fu 2023	Review
Gabryelska 2024	Lack of objective MD diagnosis
Gabryelska 2023	Poster Abstract
Gabryelska 2024	Lack of objective MD diagnosis
Gandrakota 2024	Review
Garg 2024	Conference abstract
Garrivet 2024	Lack of objective OSA diagnosis
Gawlik-Kotelnicka 2025	Lack of objective MD diagnosis
Gencdal 2025	Lack of objective MD diagnosis
Goyal 2024	Lack of objective MD diagnosis
Grandner 2023	No OSA x MD comorbidity
Gupta 2023	Lack of objective MD diagnosis
Halevi 2024	Poster Abstract
Han 2025	Lack of objective OSA diagnosis
Hartling 2025	Poster Abstract
Hein 2024	No OSA x MD comorbidity
Hou 2025	Lack of objective OSA diagnosis
Hsu 2024	Lack of objective MD diagnosis
Huang 2023	Lack of objective MD diagnosis
Huang 2025	Lack of objective MD diagnosis

Jackson 2024	Book chapter
Jackson 2020	Unclear OSA or MD diagnosis
Jacobsen 2013	No OSA x MD comorbidity
Jain 2023	Poster Abstract
Kadotani 2025	No OSA x MD comorbidity
Kallweit 2024	Poster Abstract
Kalra 2024	Lack of objective OSA diagnosis
Karakaptan 2023	Lack of objective MD diagnosis
Karkala 2024	Lack of objective MD diagnosis
Kawai 2023	Unclear comparison group
Kelly 2013	Unclear OSA or MD diagnosis
Khieu 2023	Poster Abstract
Lam 2024	Unclear OSA or MD diagnosis
Lang 2017	Lack of objective MD diagnosis
Lang 2017	Lack of objective MD diagnosis
Lee 2025	Lack of objective MD diagnosis
Lee 2016	Lack of objective MD diagnosis
Li 2023	Lack of objective MD diagnosis
Li 2023	Poster Abstract
Li 2024	Not observational study
Liao 2025	Not observational study
Liu 2024	Review
Lorrain 2025	Poster Abstract
Mangold 2025	Poster Abstract
Martinez-Moreno 2025	Lack of objective MD diagnosis
Merrill 2023	Unclear comparison group
Mi 2024	Not observational study
Mysliwiec 2013	Lack of objective MD diagnosis
Niraula 2024	Lack of objective MD diagnosis
Nokes 2023	Poster Abstract
Odenthal 2024	Lack of objective OSA diagnosis
Onen 2023	Lack of objective MD diagnosis
Okada 2022	Unclear comparison group
Palombini 2024	Poster Abstract
Parthasarathy 2023	Poster Abstract
Parthasarathy 2023	Poster Abstract
Patel 2023	Age < 18 years
Patel 2024	Poster Abstract
Pavord 2023	Conference abstract
Pepin 2024	Conference abstract
Pierobon 2023	Lack of objective MD diagnosis
Pignatiello 2023	Brief Report
Pradines 2018	No separate OSA group
Primavera 2024	Unclear comparison group

Quaglia 2024	Conference abstract
Rubina 2024	Not in English
Santiago 2024	Poster Abstract
Shaw 2024	Lack of objective MD diagnosis
Silveira 2022	Unclear comparison group
Simmons 2023	Poster Abstract
Singh 2025	Lack of objective MD diagnosis
Singh 2022	Lack of objective MD diagnosis
Soreca 2015	Brief Report
Suh 2024	Lack of objective MD diagnosis
Sultan 2023	Lack of objective OSA diagnosis
Susa 2023	Lack of objective MD diagnosis
Terry 2024	Unclear OSA or MD diagnosis
Thiesse 2023	No OSA x MD comorbidity
Thomas 2024	Lack of objective MD diagnosis
Titone 2023	Lack of objective MD diagnosis
Trzepizur 2025	Lack of objective MD diagnosis
Uzer 2020	No OSA x MD comorbidity
Vargas-Ramirez 2024	Poster Abstract
Wang 2024	Lack of objective MD diagnosis
Wang 2024	No OSA x MD comorbidity
Wan 2023	No OSA x MD comorbidity
Wan 2023	Not in English
Weber 2024	Poster Abstract
Wickwire 2024	Lack of objective MD diagnosis
Wickwire 2023	Unclear OSA or MD diagnosis
Wickwire 2024	Poster Abstract
Wickwire 2024	Unclear comparison group
Wickwire 2023	Conference abstract
Wickwire 2023	Conference abstract
Wohlgemuth 2025	Poster Abstract
Wohlgemuth 2023	Conference abstract
Wongchan 2023	Conference abstract
Wu 2024	Unclear OSA or MD diagnosis
Xu 2023	Lack of objective MD diagnosis
Zhai 2024	Not in English
Zhang 2025	Unclear OSA or MD diagnosis
Zhang 2024	Lack of objective MD diagnosis
Zhao 2024	Review
Zhao 2023	Lack of objective MD diagnosis
Zhu 2025	Review
Zolfaghari 2024	Lack of objective OSA diagnosis

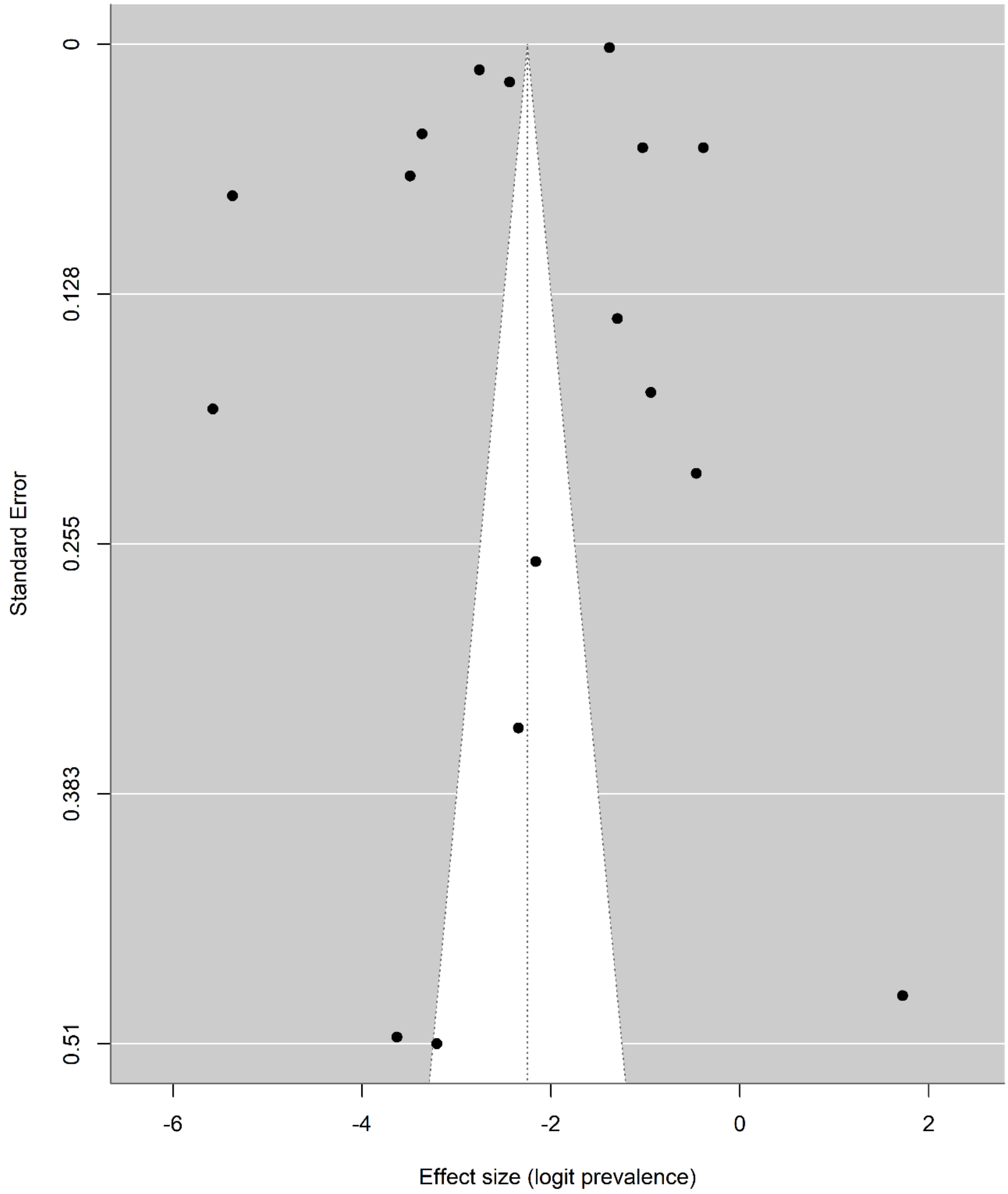

Supplementary Figure S1: Funnel plot of the mood disorder prevalence in the OSA population

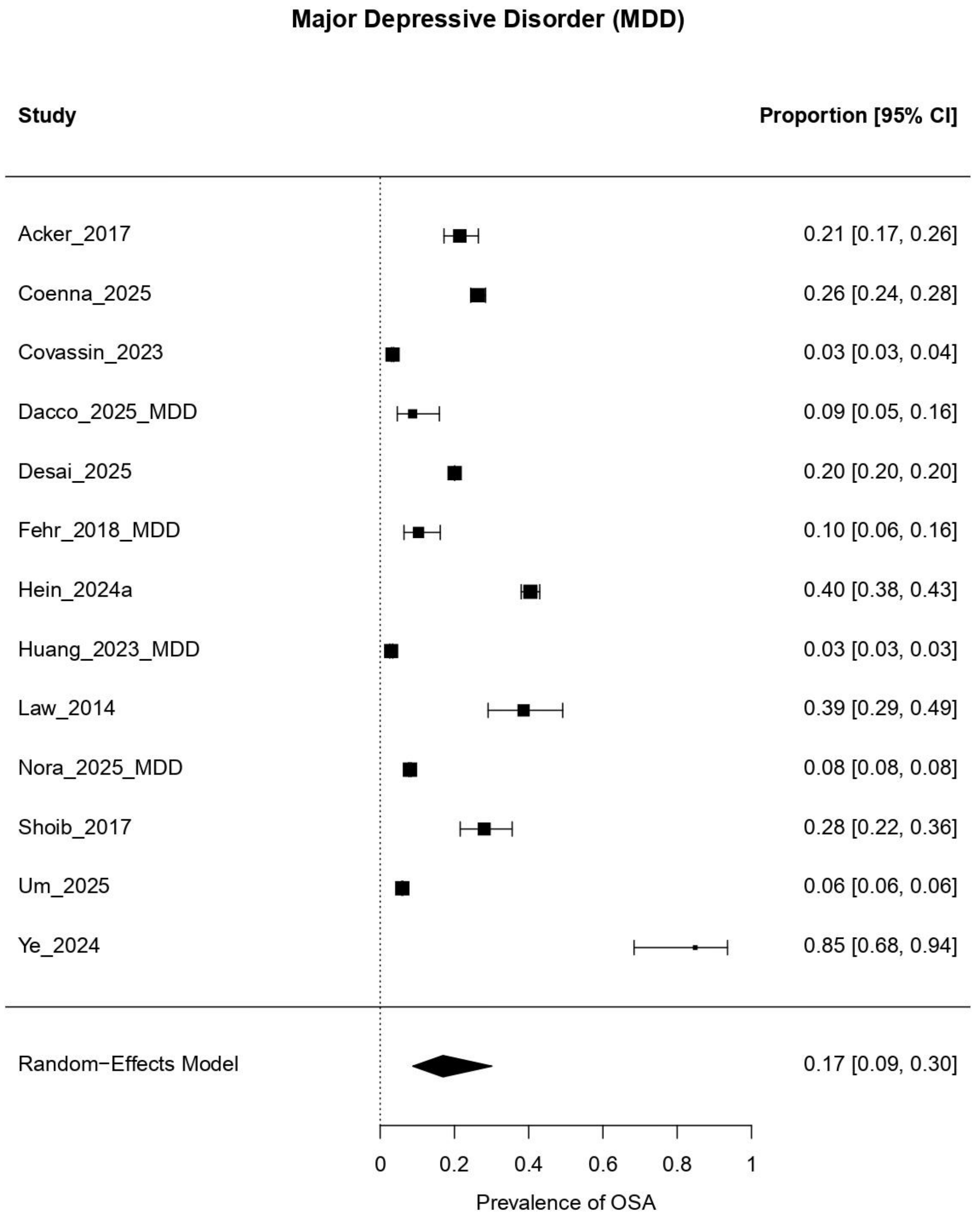

Supplementary Figure S2: Forest plot of MDD in mood disorder prevalence in the OSA population

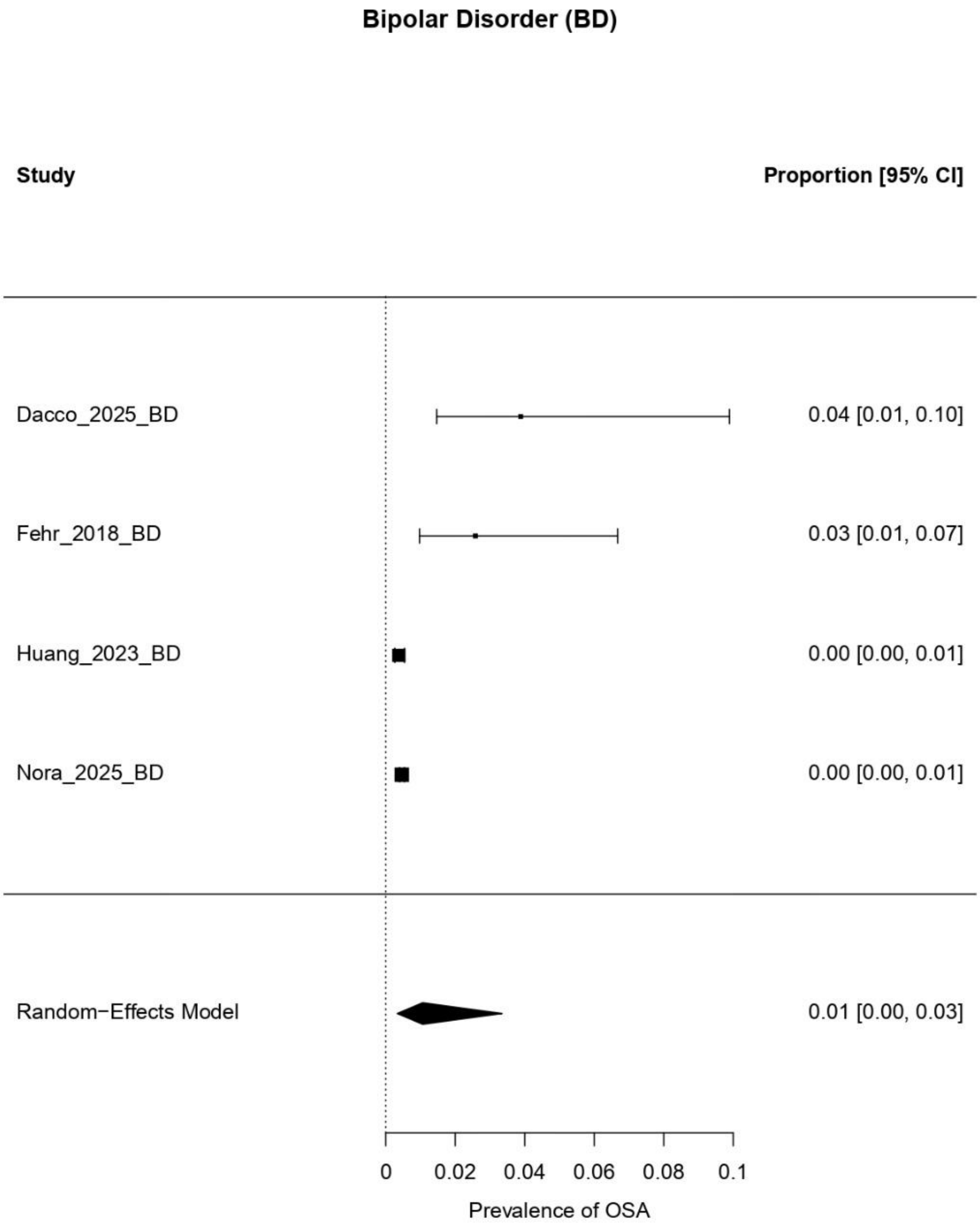

Supplementary Figure S3: Forest plot of BD in mood disorder prevalence in the OSA population

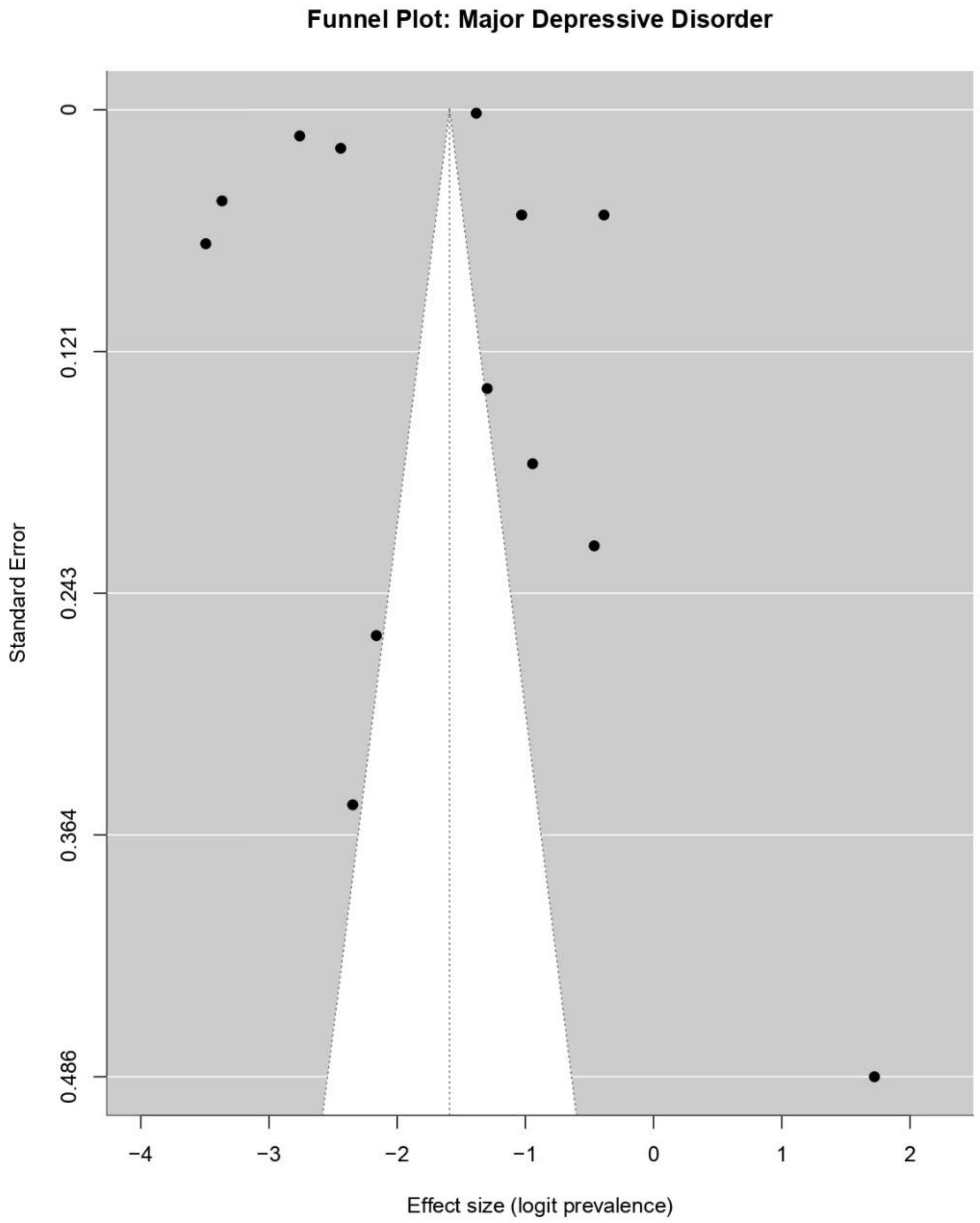

Supplementary Figure S4: Funnel plot of MDD in mood disorder prevalence in the OSA population

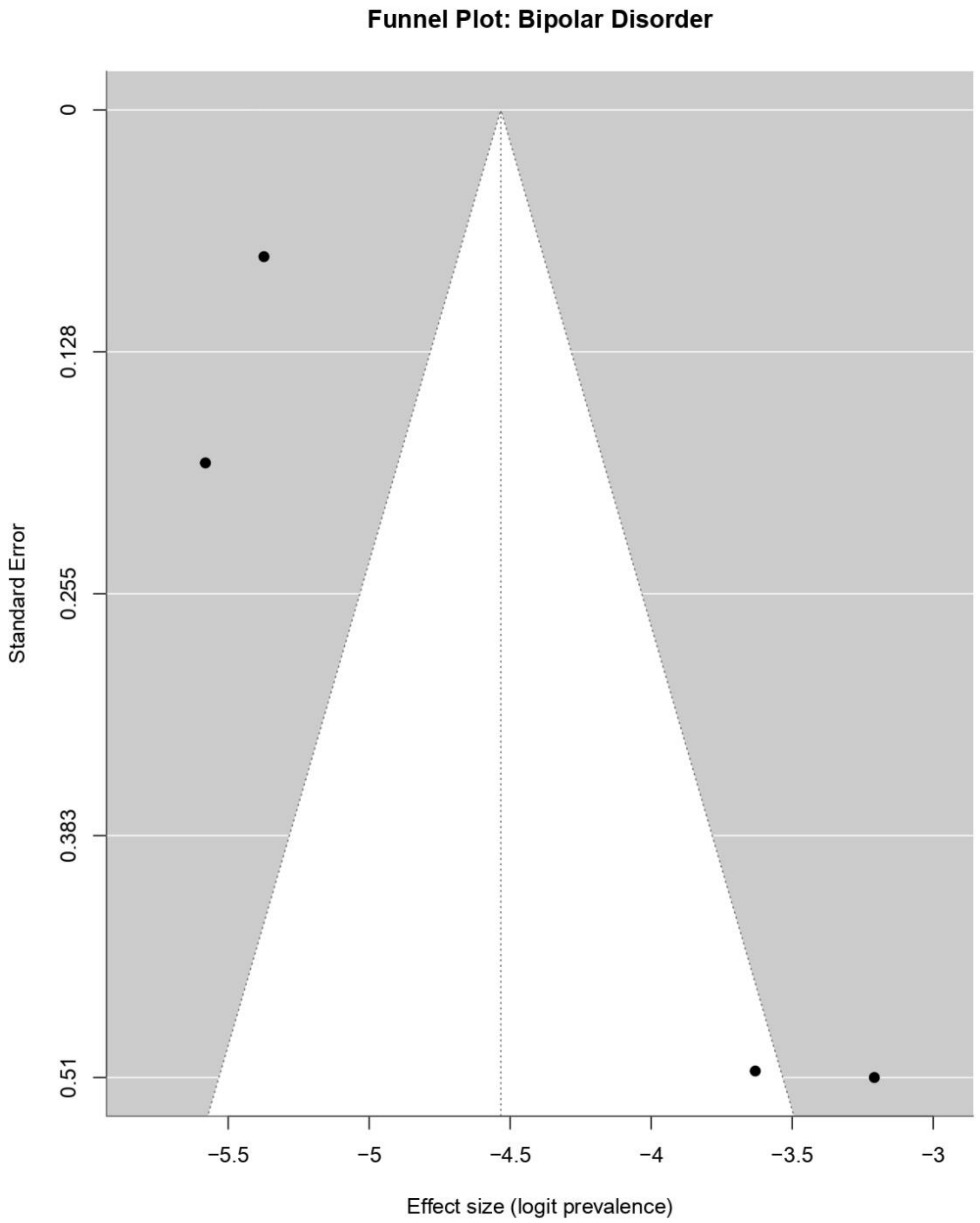

Supplementary Figure S5: Funnel plot of BD in mood disorder prevalence in the OSA population

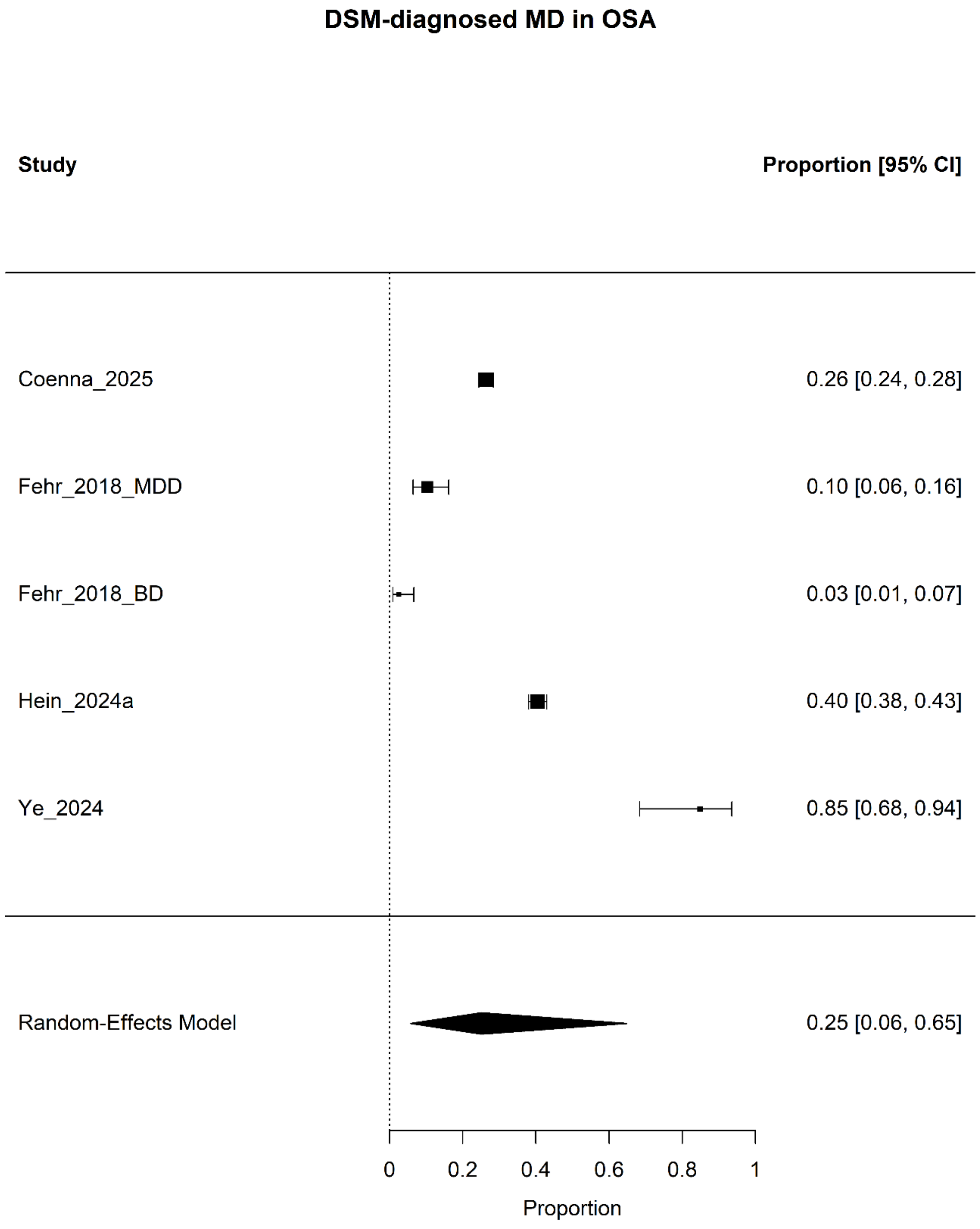

Supplementary Figure S6: Forest plot of DSM/SCID in mood disorder prevalence in the OSA population

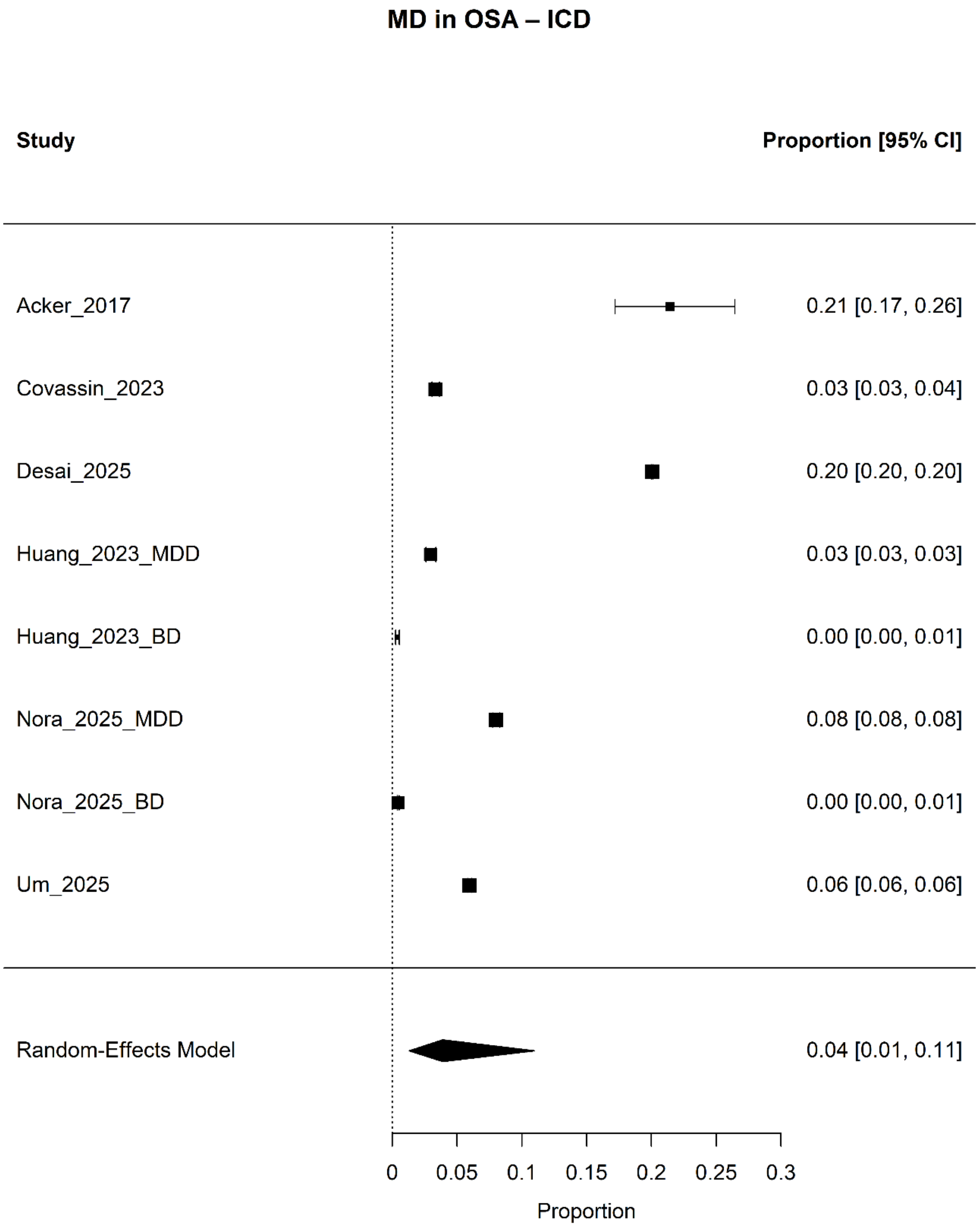

Supplementary Figure S7: Forest plot of ICD in mood disorder prevalence in the OSA population

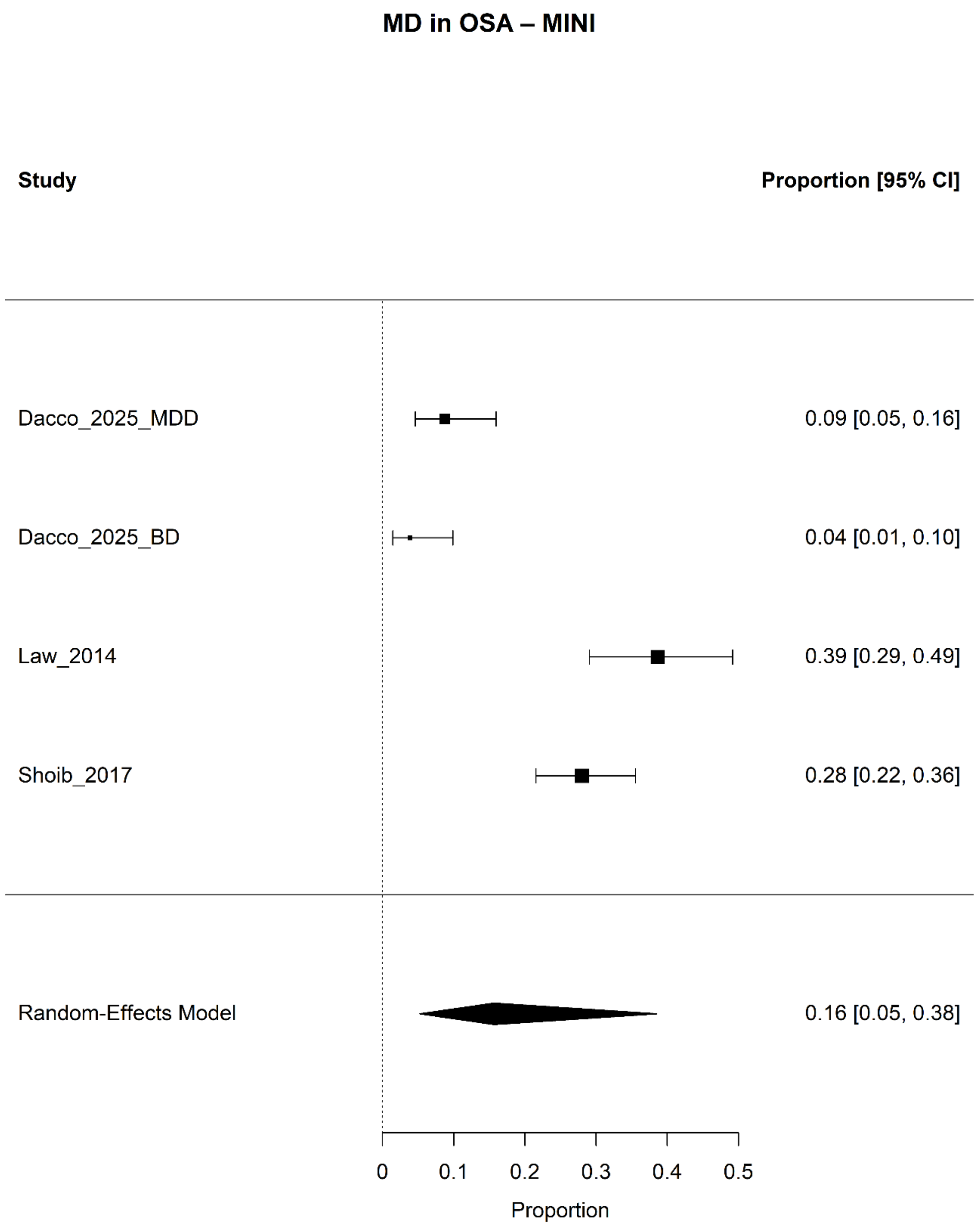

Supplementary Figure S8: Forest plot of MINI in mood disorder prevalence in the OSA population

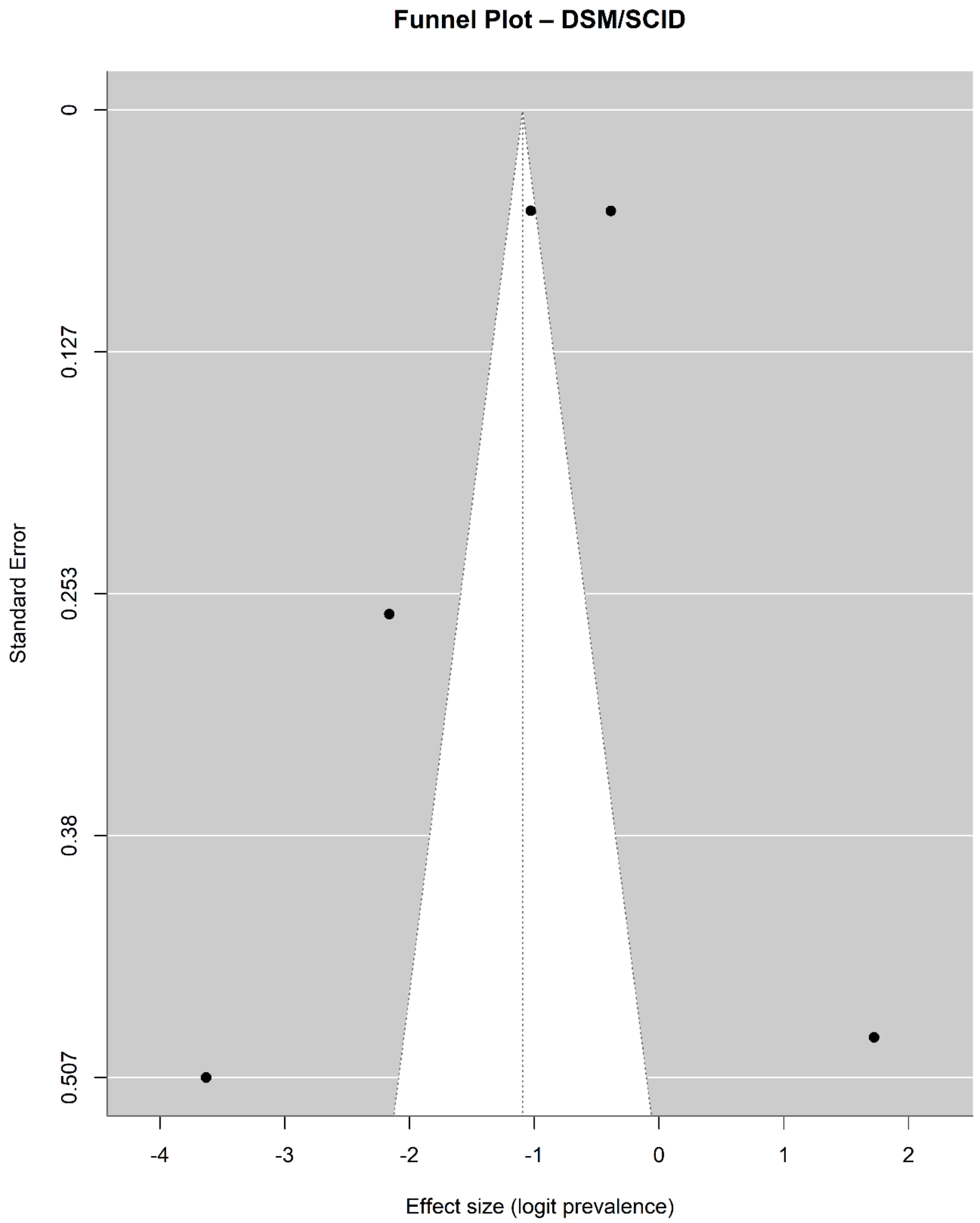

Supplementary Figure S9: Funnel plot of DSM/SCID in mood disorder prevalence in the OSA population

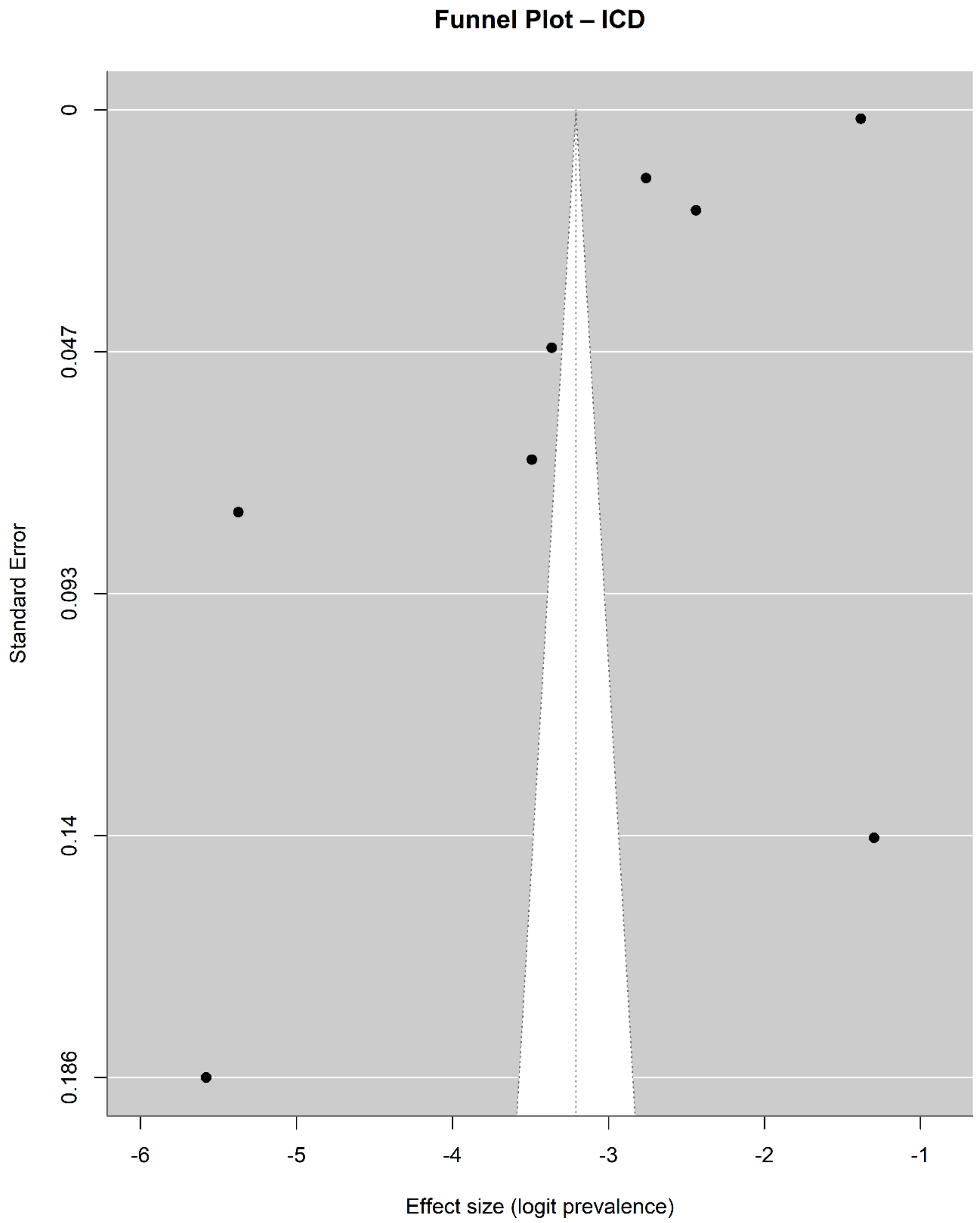

Supplementary Figure S10: Funnel plot of ICD in mood disorder prevalence in the OSA population

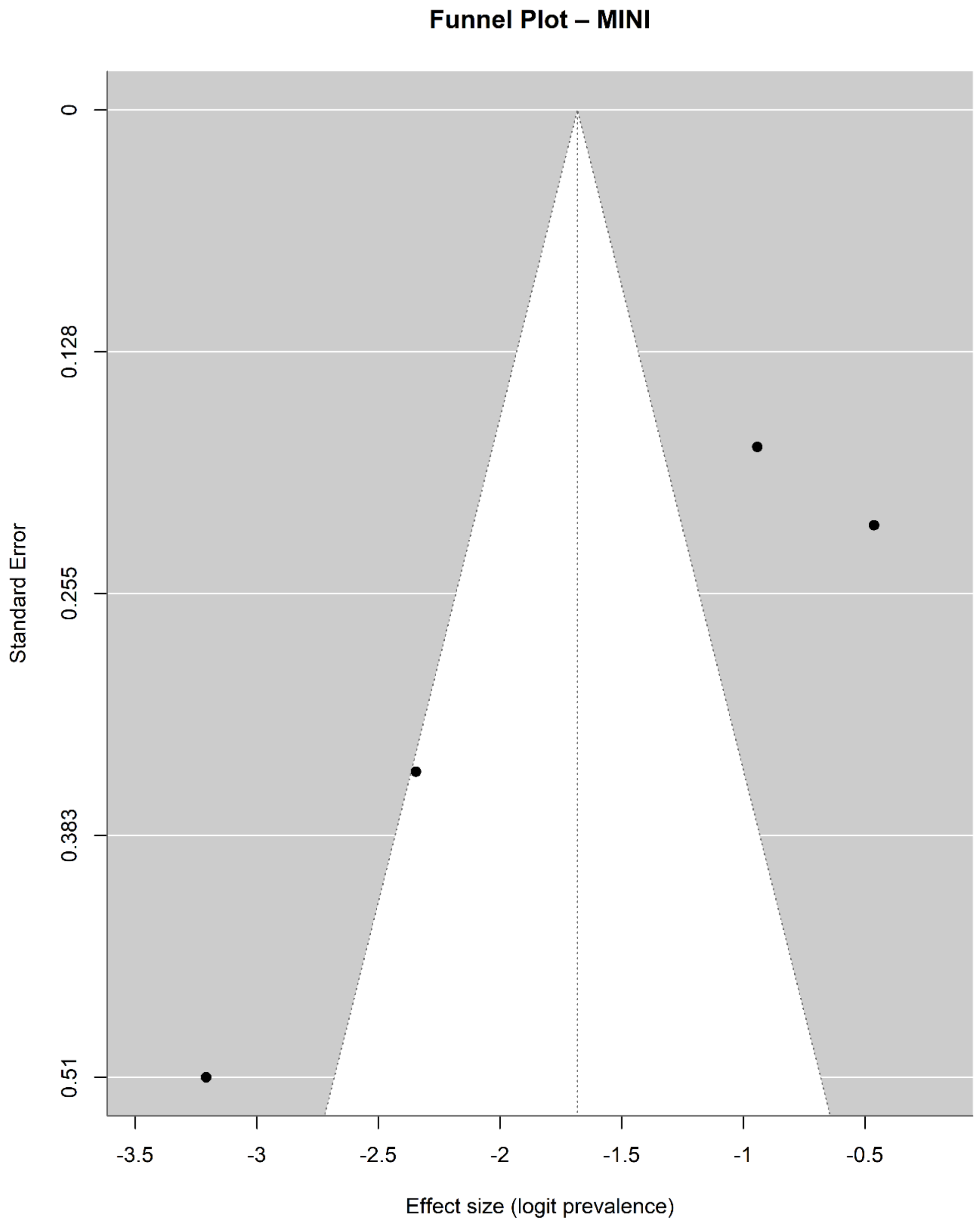

Supplementary Figure S11: Funnel plot of MINI in mood disorder prevalence in the OSA population

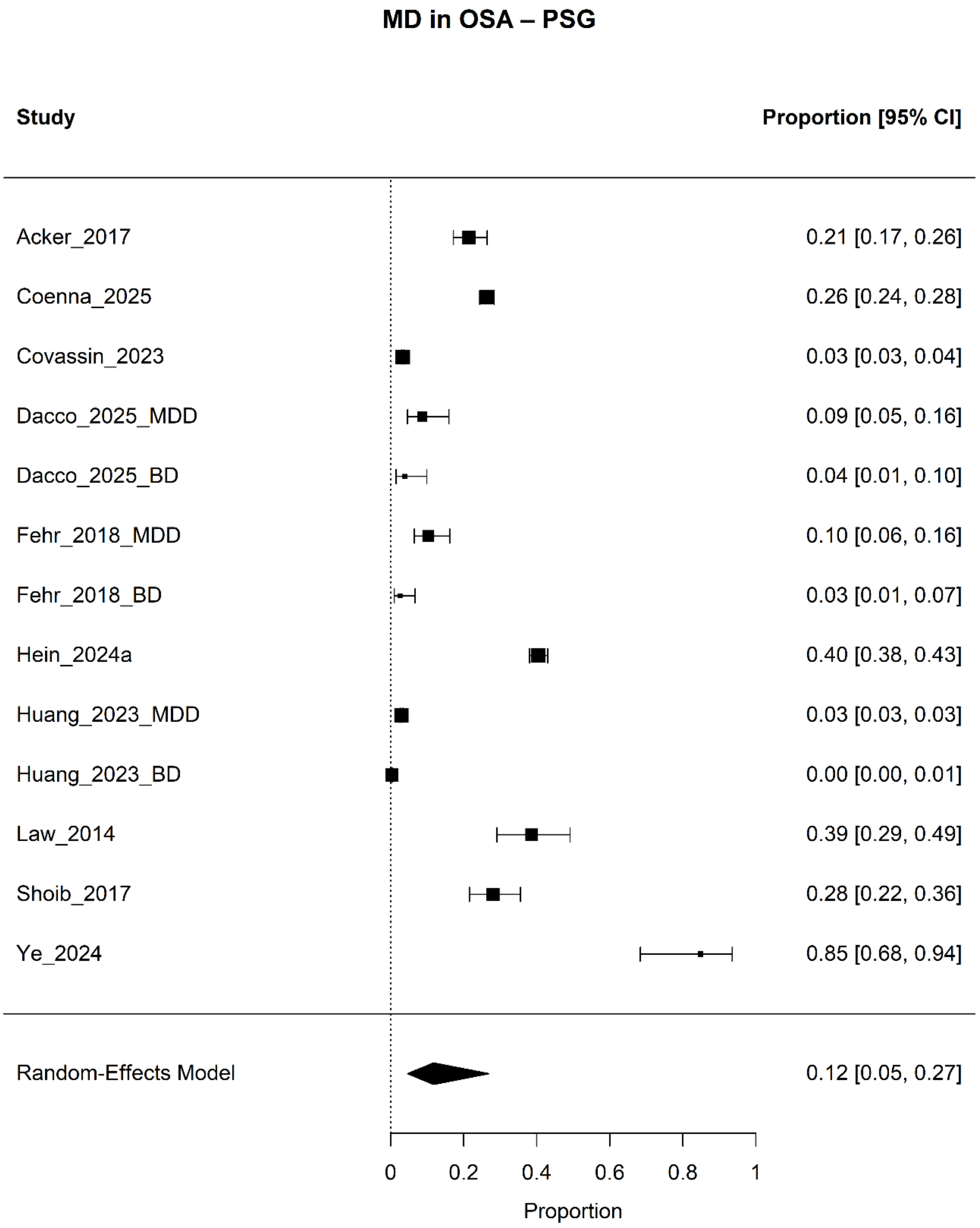

Supplementary Figure S12: Forest plot of PSG in mood disorder prevalence in the OSA population

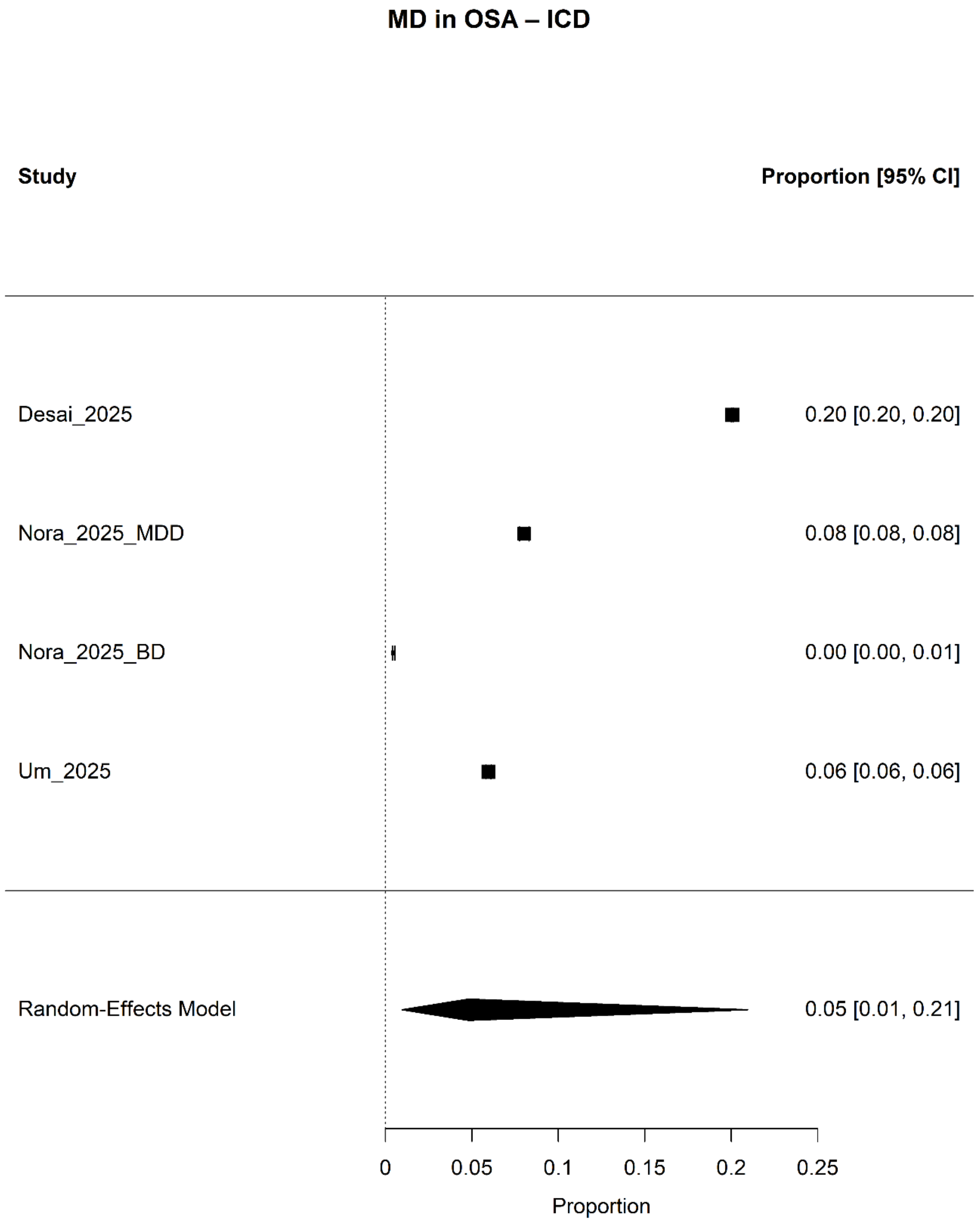

Supplementary Figure S13: Forest plot of ICD in mood disorder prevalence in the OSA population

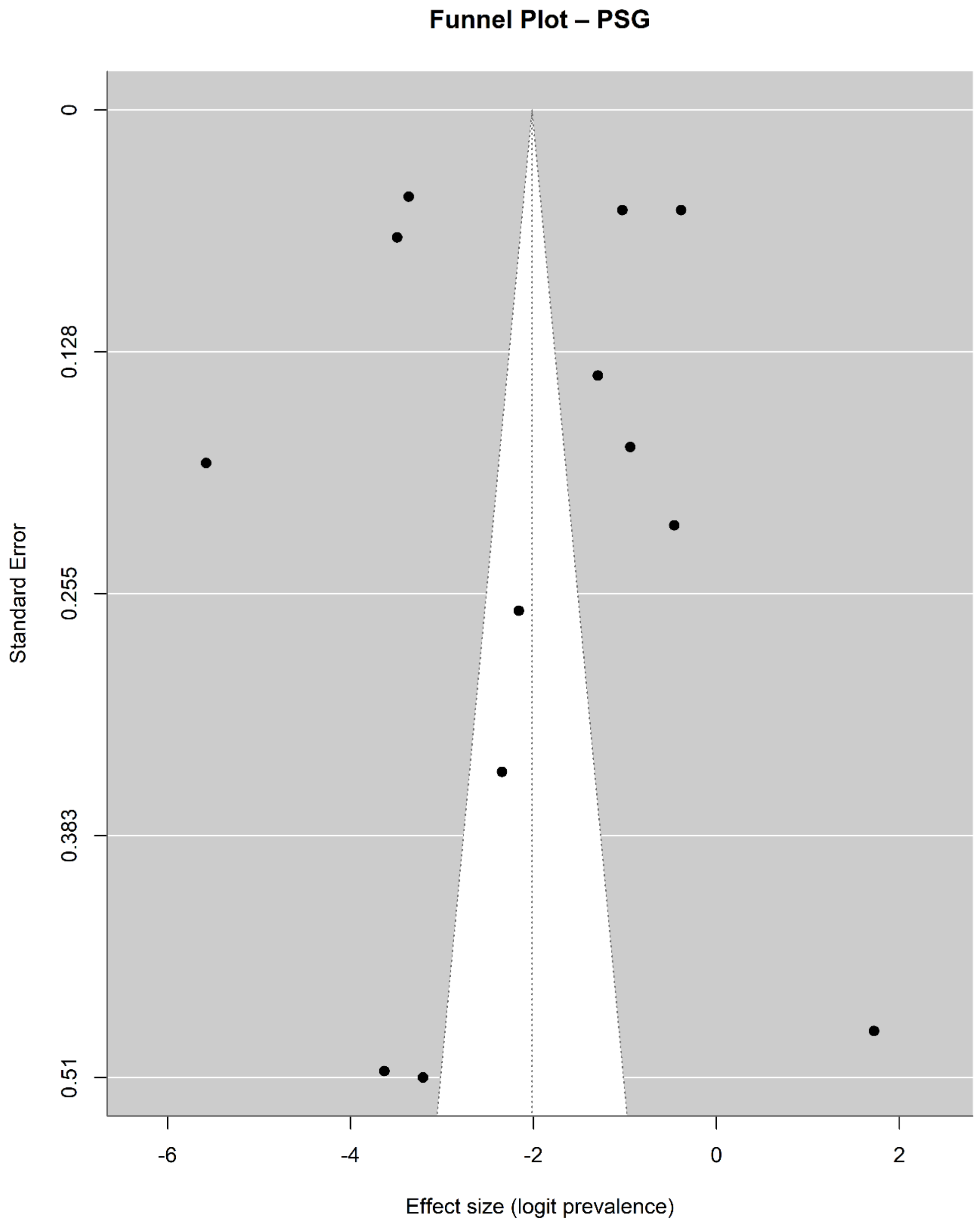

Supplementary Figure S14: Funnel plot of PSG in mood disorder prevalence in the OSA population

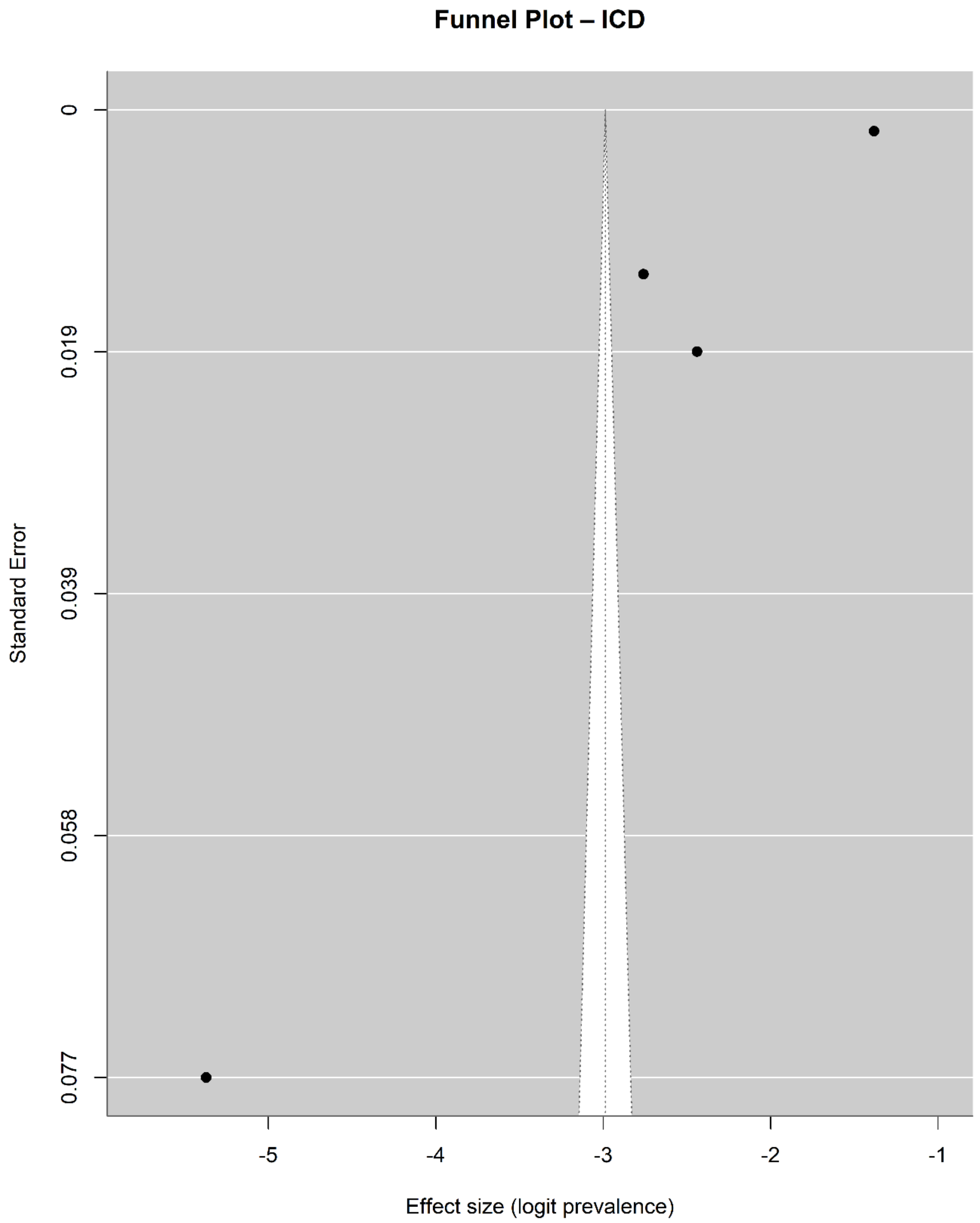

Supplementary Figure S15: Funnel plot of ICD in mood disorder prevalence in the OSA population

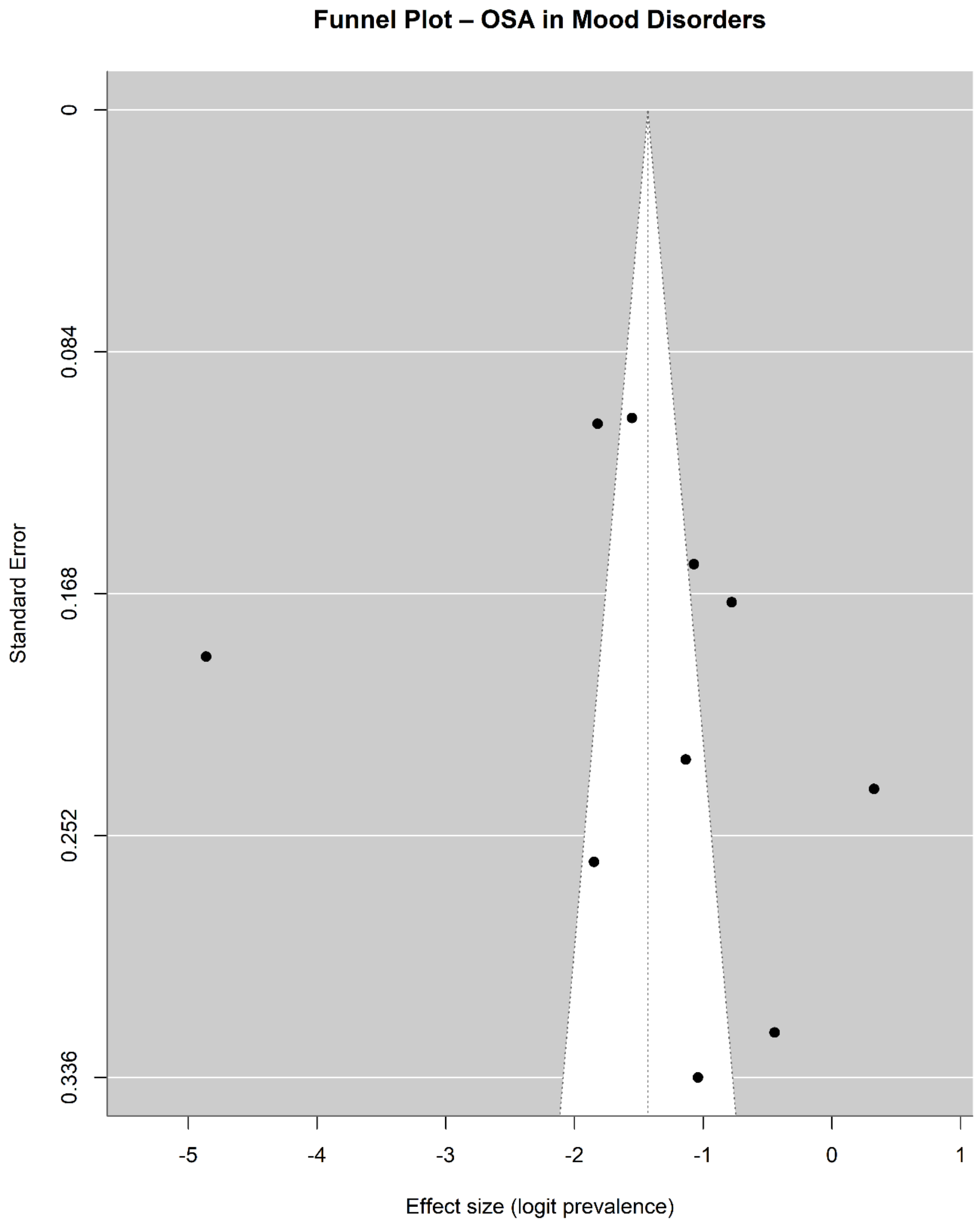

Supplementary Figure S16: Funnel plot of the OSA prevalence in the mood disorder population

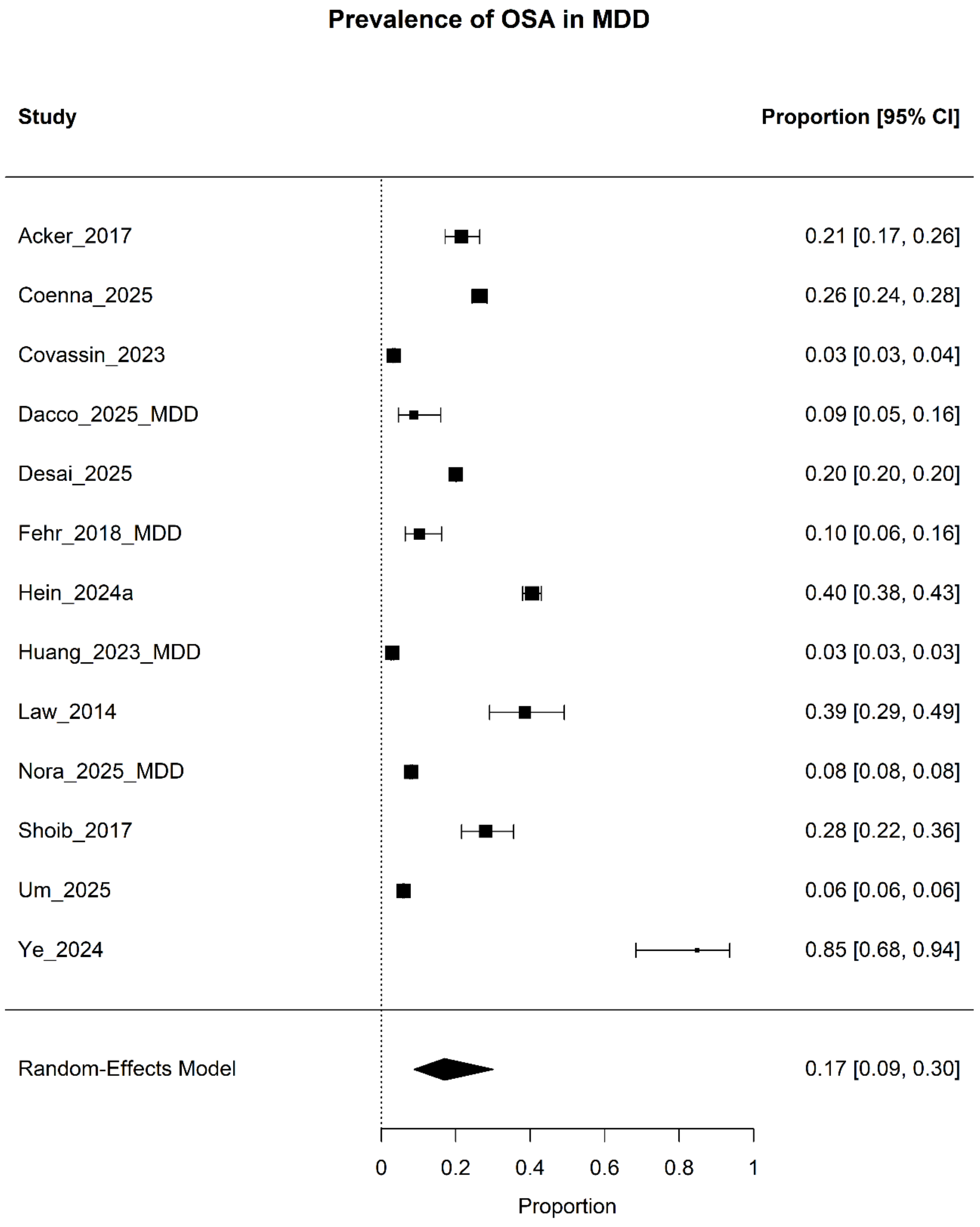

Supplementary Figure S17: Forest plot of MDD in OSA prevalence in the mood disorder population

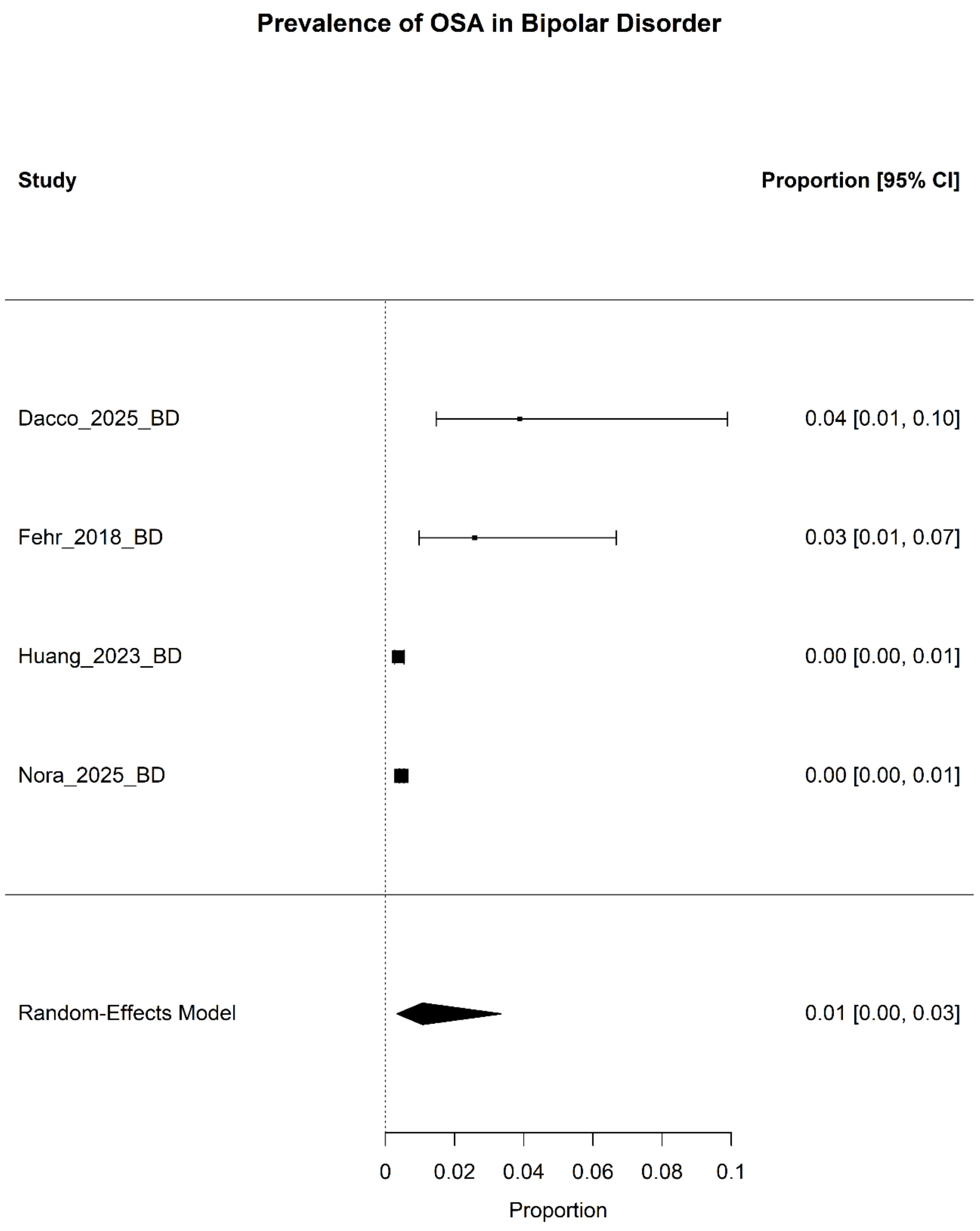

Supplementary Figure S18: Forest plot of BD in OSA prevalence in the mood disorder population

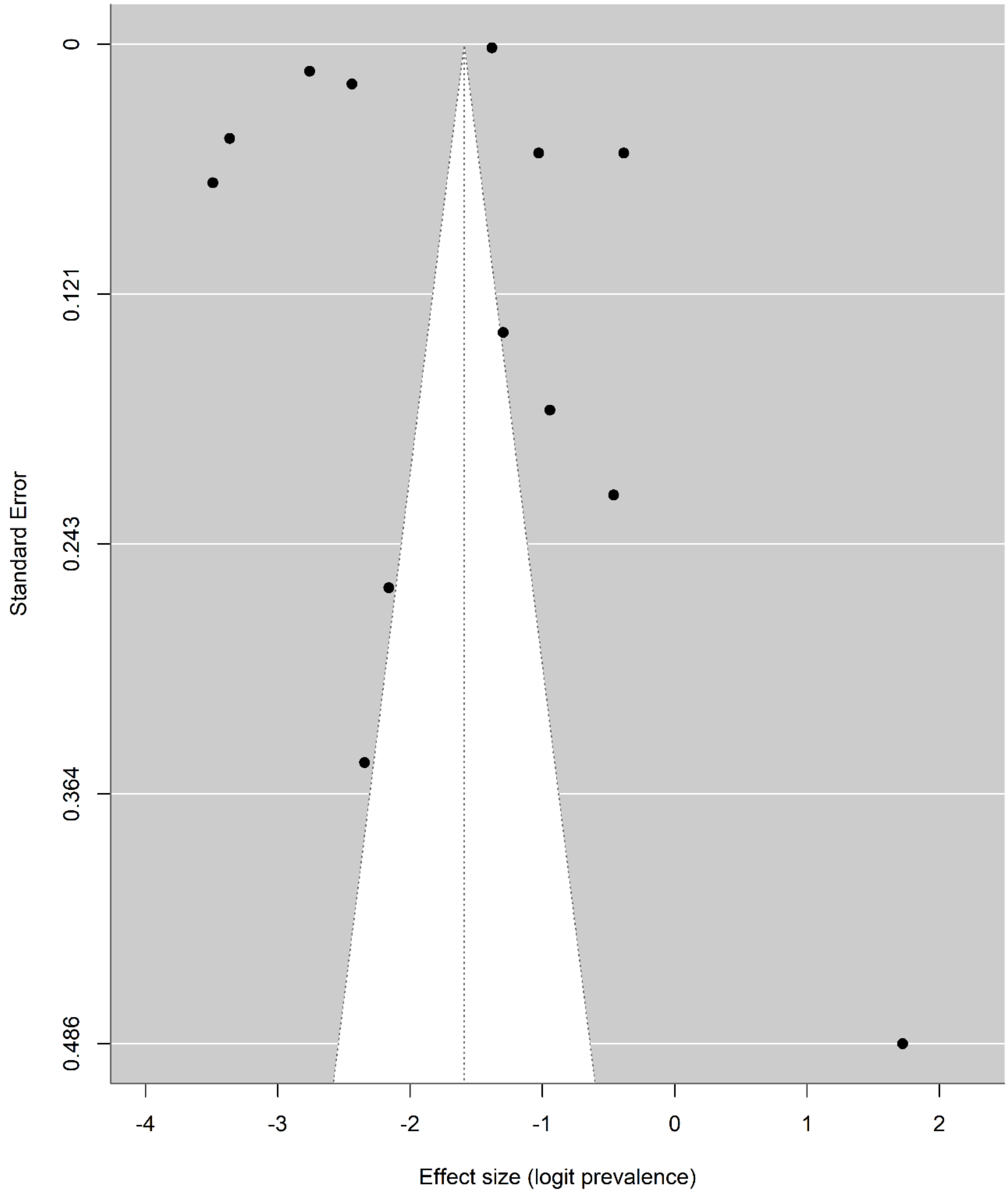

Supplementary Figure S19: Funnel plot of MDD in OSA prevalence in the mood disorder population

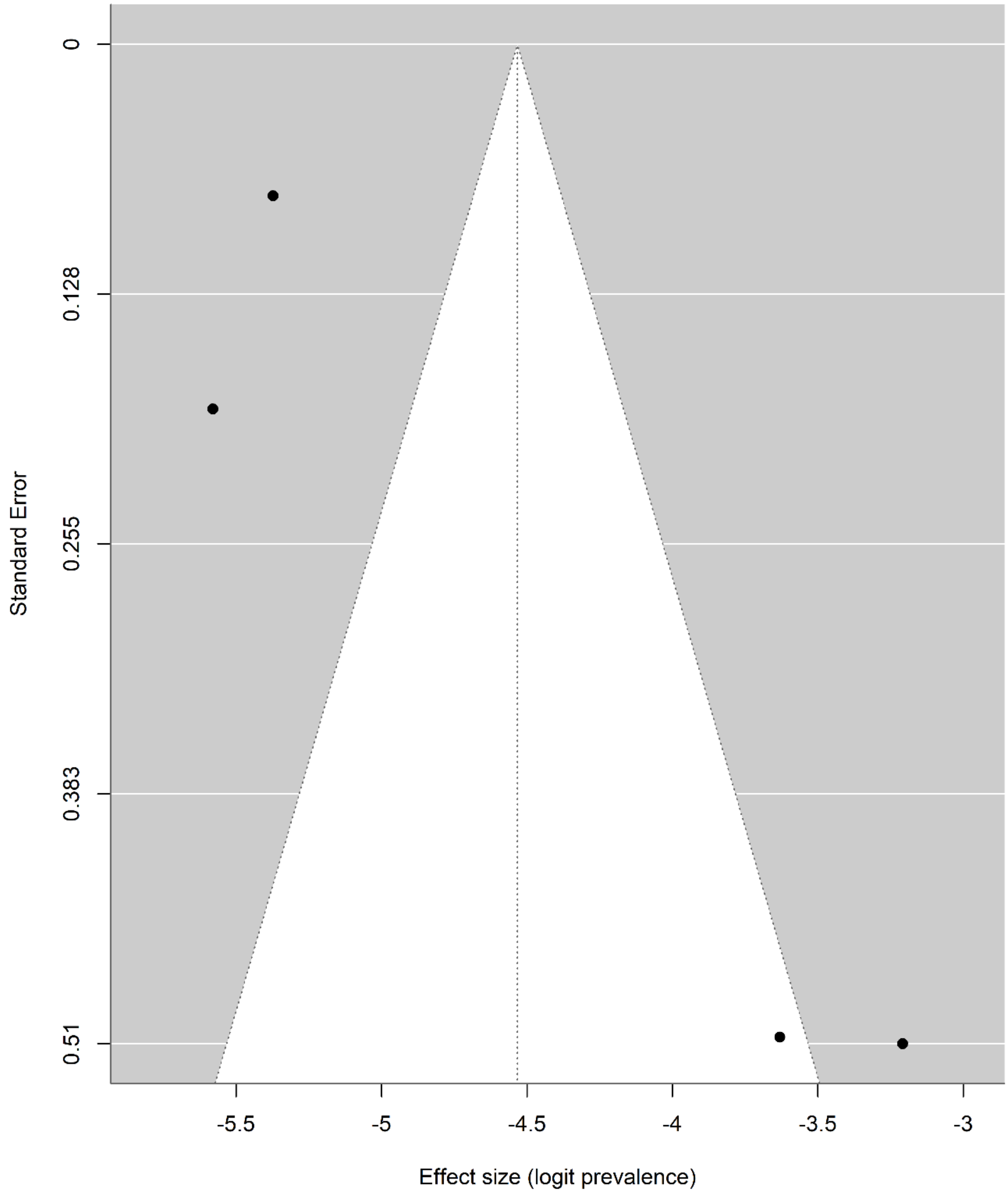

Supplementary Figure S20: Funnel plot of BD in OSA prevalence in the mood disorder population

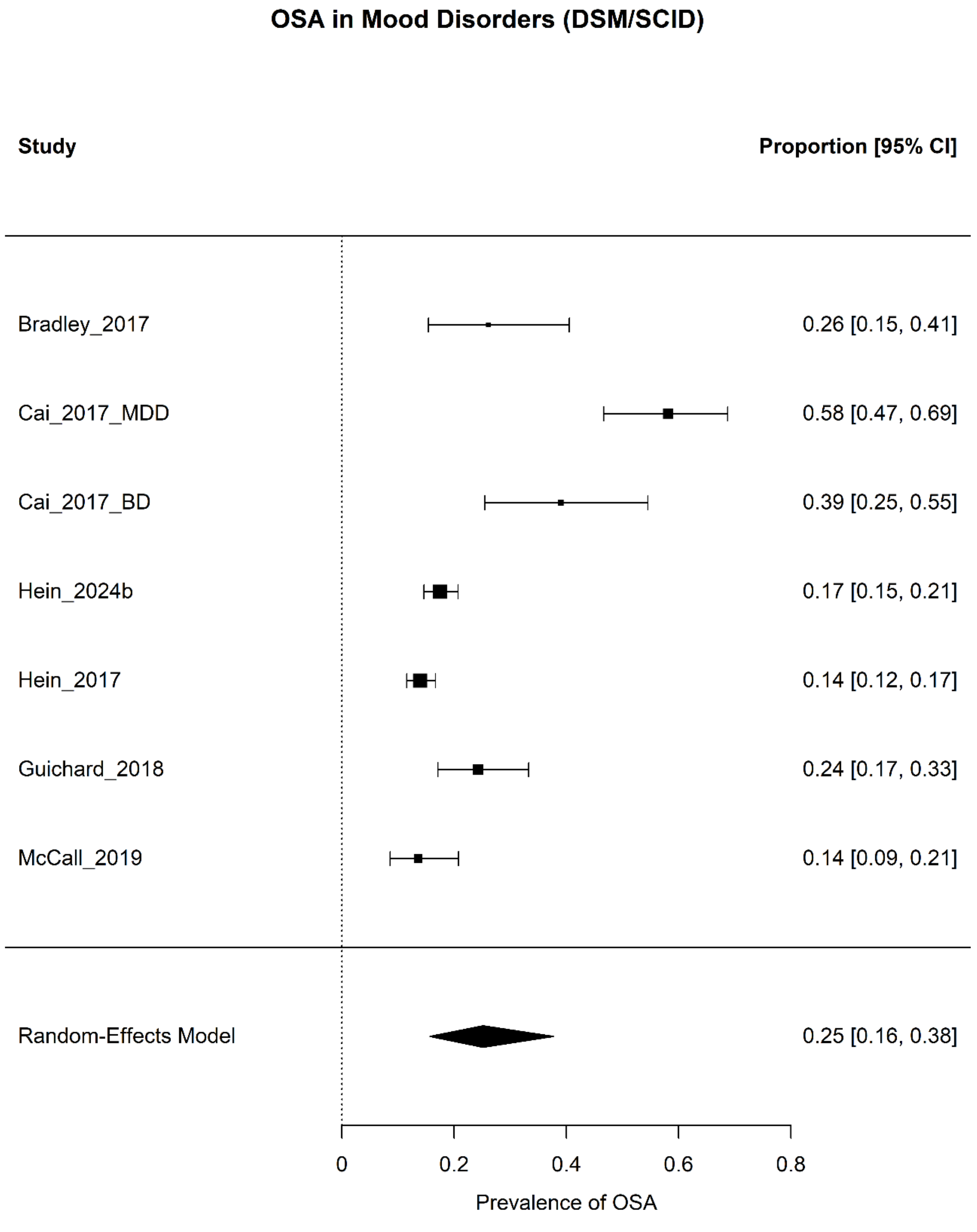

Supplementary Figure S21: Forest plot of DSM/SCID in OSA prevalence in the mood disorder population

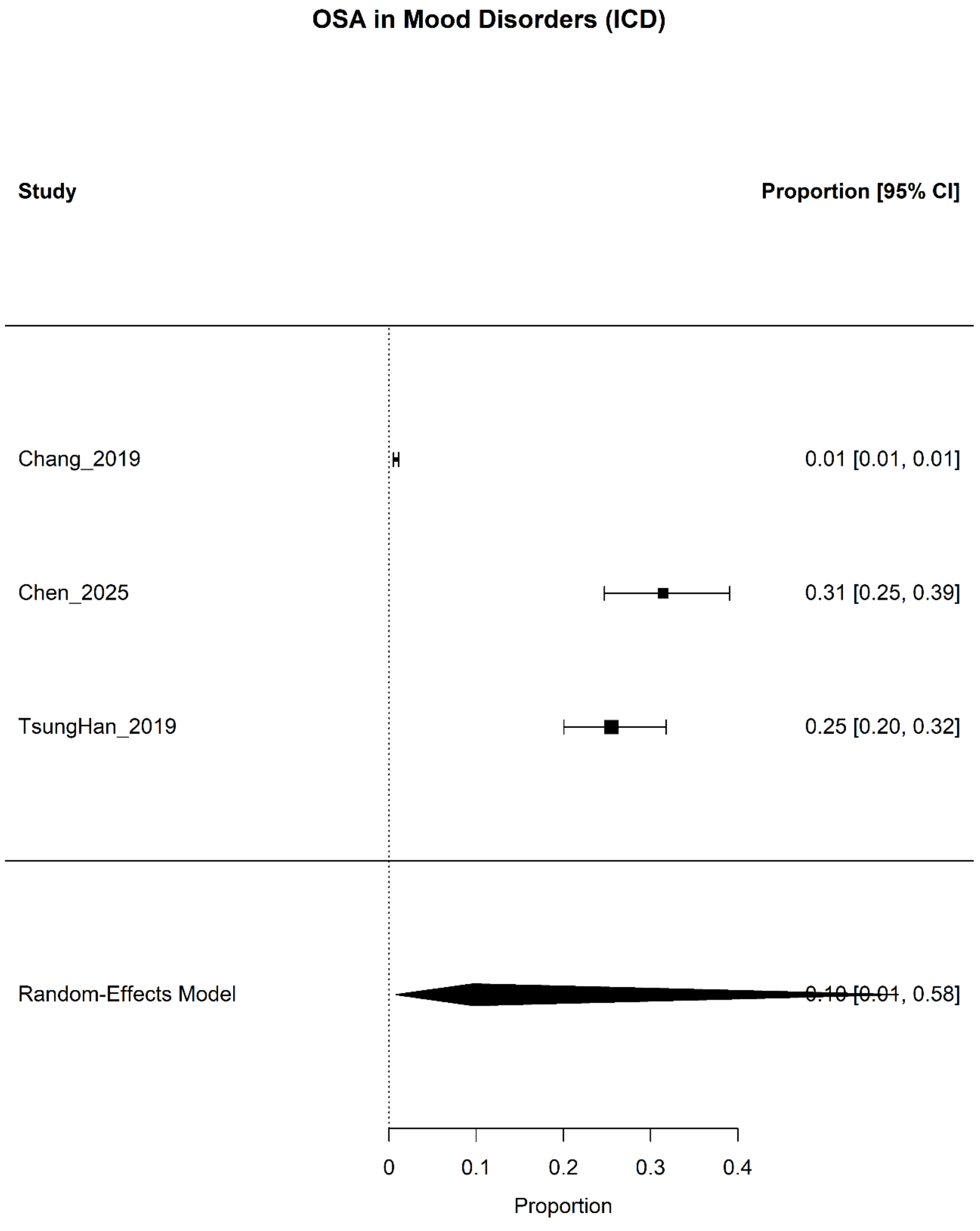

Supplementary Figure S22: Forest plot of ICD in OSA prevalence in the mood disorder population

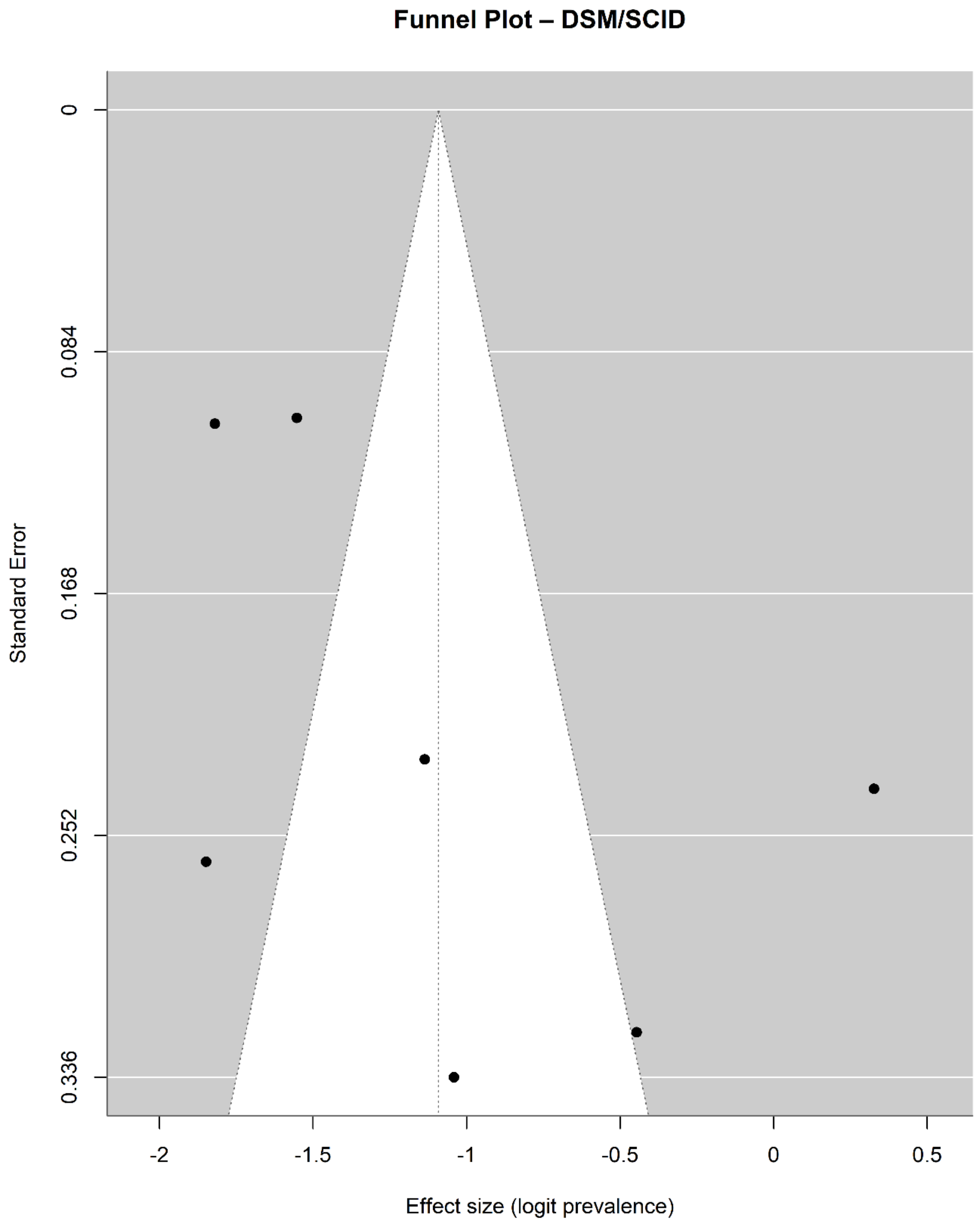

Supplementary Figure S23: Funnel plot of DSM/SCID in OSA prevalence in the mood disorder population

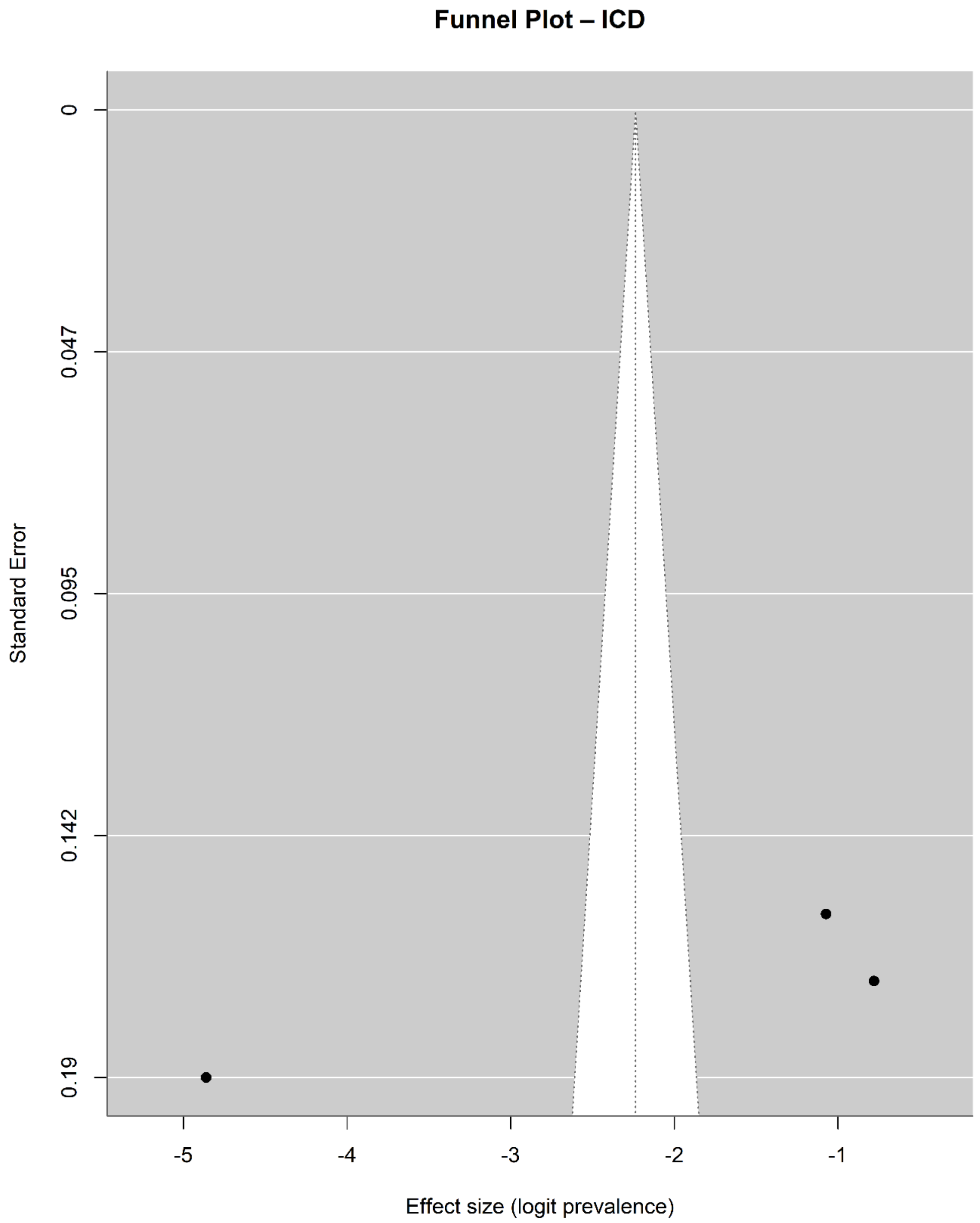

Supplementary Figure S24: Funnel plot of ICD in OSA prevalence in the mood disorder population

Supplementary Table S3: Results of meta-analyses

Analysis	Number of analyzed groups	Pooled Prevalance (95% CI)	I²	Q-test	Q-test p	Bias p-value
**MD in OSA**						
**Overall**	17	0.0954 (0.0395 to 0.2128)	99.90%	20131.25	<0.001	0.06
**MD group**						
MDD	13	0.1692 (0.0879 to 0.3008)	99.97%	16980.87	<0 .0001	**0.03**
BD	4	0.0106 (0.0033 to 0.0334)	96.42%	30.68	< 0.0001	NA
**Diagnosis of MD**						
DSM/SCID	5	0.2509 (0.0575 to 0.6477)	99.72%	161.95	< 0.0001	NA
ICD	8	0.0388 (0.0131 to 0.1096)	99.99%	19580.43	< 0.0001	NA
MINI	4	0.1568 (0.0524 to 0.3848)	95.11%	39.11	< 0.0001	NA
**Diagnosis of OSA**						
PSG	13	0.1175 (0.0460 to 0.2686)	99.72%	3210.99	< 0.0001	0.8833
ICD	4	0.0480 (0.0095 to 0.2095)	99.99%	16309.56	< 0.0001	NA
**OSA in MD**						
**Overall**	10	0.1931 (0.0925 to 0.3598)	98.34%	422.08	<0.0001	0.4385
**MD group**						
MDD	7	0.2434 (0.1568 to 0.3574)	95.28%	91.73	<0.0001	NA
BD	3	0.1064 (0.0078 to 0.6445)	98.67%	192.11	<0.0001	NA
**Diagnosis of MD**						
DSM/SCID	7	0.2513 (0.1569 to 0.3771)	94.47%	84.85	<0.0001	NA
ICD	3	0.0965 (0.0081 to 0.5838)	99.43%	313.29	<0.0001	NA
MINI	0	NA	NA	NA	NA	NA
**Diagnosis of OSA**						
PSG	10	0.1931 (0.0925 to 0.3598)	98.34%	422.0757	<0.0001	0.4385
ICD	0	NA	NA	NA	NA	NA

Supplementary Table S4: Results of meta-regression analyses

Analysis	No. of studies	β	95% CI Lower	95% CI Upper	p-value
**Mood Disorders in OSA**					
Female %	11	-0.0426	-0.1067	0.0214	0.1923
Mean age	12	-0.0839	-0.1728	0.0050	0.0643
Mean BMI	8	-0.4472	-0.9232	0.0289	0.0656
Mean AHI	7	0.0761	-0.0234	0.1757	0.1340
NOS score	13	-0.3535	-1.4200	0.7131	0.5160
**OSA in Mood Disorders**					
Female %	9	0.0566	-0.0626	0.1757	0.3524
Mean age	9	0.1820	0.0308	0.3332	0.0183
Mean BMI	8	-0.1806	-0.3217	-0.0396	0.0121
Mean AHI	7	0.0299	-0.0515	0.1113	0.4709
NOS score	9	-0.4487	-3.5723	2.6750	0.7783

*Bold text indicates statistical significancy.
